# Mechanical Force‐Induced cGAS Activation in Carcinoma Cells Facilitates Splenocytes into Liver to Drive Metastasis

**DOI:** 10.1002/advs.202401127

**Published:** 2024-12-31

**Authors:** Xurui Zhang, Na Huang, Yanhua Mu, Haiyan Chen, Mengchen Zhu, Shaoying Zhang, Pengfei Liu, Hailong Zhang, Huan Deng, Keping Feng, Qi Shang, Xi Liu, Chen Zhang, Mengjiao Shi, Lan Yang, Jin Sun, Guangyao Kong, Jing Geng, Shemin Lu, Zongfang Li

**Affiliations:** ^1^ Department of General Surgery National‐Local Joint Engineering Research Center of Biodiagnostic & Biotherapy The Second Affiliated Hospital of Xi'an Jiaotong University Xi'an Shaanxi 710004 China; ^2^ Shaanxi Provincial Clinical Medical Research Center for Liver and Spleen Diseases CHESS‐Shaanxi consortium The Second Affiliated Hospital of Xi'an Jiaotong University Xi'an Shaanxi 710004 China; ^3^ Shaanxi International Cooperation Base for Inflammation and Immunity Shaanxi Provincial Academician Workstation The Second Affiliated Hospital of Xi'an Jiaotong University Xi'an Shaanxi 710004 China; ^4^ Key Laboratory of Environment and Genes Related to Diseases Xi'an Jiaotong University Ministry of Education of China Xi'an Shaanxi 710061 China

**Keywords:** liver metastasis, splenocytes, mechanical force, nuclear deformation, cGAS activation

## Abstract

Liver metastasis is the main cause of cancer‐related mortality. During the metastasis process, circulating carcinoma cells hardly pass through narrow capillaries, leading to nuclear deformation. However, the effects of nuclear deformation and its underlying mechanisms on metastasis need further study. Here, it is shown that mechanical force‐induced nuclear deformation exacerbates liver metastasis by activating the cGAS‐STING pathway, which promotes splenocyte infiltration in the liver. Mechanical force results in nuclear deformation and rupture of the nuclear envelope with inevitable DNA leakage. Cytoplasmic DNA triggers the activation of cGAS‐STING pathway, enhancing the production of IL6, TNFα, and CCL2. Additionally, splenocyte recruitment by the proinflammatory cytokines support carcinoma cell survival and colonization in the liver. Importantly, both intervening activity of cGAS and blocking of splenocyte migration to the liver efficiently ameliorate liver metastasis. Overall, these findings reveal a mechanism by which mechanical force‐induced nuclear deformation exacerbates liver metastasis by regulating splenocyte infiltration into the liver and support targeting cGAS and blocking splenocyte recruitment as candidate therapeutic approaches for liver metastasis.

## Graphical abstract

This study reports that mechanical force‐induced nuclear deformation in capillaries leads to cGAS activation in carcinoma cells which exacerbates liver metastasis via facilitating splenocyte migration into liver. Intervening of cGAS activation and blocking splenocyte infiltration both ameliorate liver metastasis.

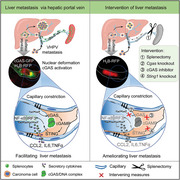

## 1 Introduction

Metastasis is the major feature of malignancies leading to cancer‐related deaths and the process by which tumor cells spread from a primary tumor site to distant locations. The liver is the most common organ for metastasis, e.g., melanoma, pancreatic and colorectal cancer manifest high metastasis to the liver.^[^ ^1^, ^2^ ^]^ The investigations of pathology and anatomy indicate that the metastasis of these cancers from the primary site to the liver is predominantly via the hepatic portal vein (HPV) system.^[^ ^1^ ^]^ Although this metastatic tropism reflects mechanical trapping of circulating tumor cells,^[^ ^3^ ^]^ liver metastasis is dependent on the formation of a pro‐metastatic niche that supports the carcinoma cell to colonization and outgrowth.^[^ ^4^, ^5^, ^6^ ^]^ However, more attention should be paid to exploring the response of carcinoma cells to mechanical force in the circulation. Since the carcinoma cells via HPV blood represent the initial status to colonize in liver, blocking the colonization progress may potentially interrupt the way of liver metastasis.

The complex metastatic cascade can be simplified into two major phases: the translocation phase and the colonization phase. In the translocation phase, carcinoma cells encounter interstitial spaces of the order of 0.1–20 µm in diameter and are ultimately arrested in capillaries with a luminal inner diameter of 6–9 µm, which is smaller than the nuclear size of most carcinoma cells.^[^ ^7^, ^8^, ^9^, ^10^ ^]^ The migration through such confined environments puts considerable mechanical stress on the cell nucleus and leads to different outcomes. The mild nuclear deformation induces tension of nuclear envelope (NE) and promotion of import/export through nuclear pores.^[^ ^11^ ^]^ The moderate nuclear deformation induces transient NE rupture that allows uncontrolled exchange between the nucleoplasm and cytoplasm, and then the exposure of genomic DNA to cytoplasm leads to both DNA damage and activation of the cyclic GMP‐AMP synthase (cGAS), a cytoplasmic DNA sensor.^[^ ^12^, ^13^, ^14^, ^15^ ^]^ The severe nuclear deformation frequently experiences NE rupture and cell death,^[^ ^16^, ^17^ ^]^ which might be responsible for a highly inefficient process of carcinoma metastasis.^[^ ^18^, ^19^ ^]^ The acquired DNA damage caused by the mechanical force increases genomic instability in carcinoma cells,^[^ ^14^, ^15^, ^20^, ^21^ ^]^ but whether the cGAS activation exerts any crucial role in metastasis of carcinoma cells is an exciting and inevitable issue.

cGAS senses cytosolic double‐stranded DNA (dsDNA) regardless of intracellular or extracellular sources.^[^ ^22^, ^23^, ^24^ ^]^ Cytosolic dsDNA directly interacts with cGAS and triggers cGAS catalytic activity, which produces the second messenger 2′3′‐cyclic GMP‐AMP (cGAMP). cGAMP directly binds to the stimulator of interferon genes (STING), which then translocate to the ER‐Golgi intermediate compartment to recruit TANK‐binding kinase 1 (TBK1). Interferon regulatory factor 3 (IRF3) and nuclear factor κB (NF‐κB), as transcription factors activated by TBK1, lead to the expression of genes for inflammatory cytokines, chemokines, and type I interferons (IFNs).^[^ ^24^, ^25^, ^26^, ^27^ ^]^

At present, cGAS pathway activation has been paid great attention to the role of physiology and pathology. It has been reported that the cGAMP from cGAS activation in human and mouse breast and lung cancer cells transports to astrocytes through carcinoma–astrocyte gap junctions, which stimulates STING activation in astrocytes to produce interferon‐α (IFNα) and tumor necrosis factor (TNFα) to promote the brain metastasis.^[^ ^28^ ^]^ Another report is that high chromosomal instability (CIN) can activate the cGAS pathway to facilitate cancer metastasis by sustaining a carcinoma cell‐autonomous response to cytosolic DNA.^[^ ^14^ ^]^ Further exploration revealed that high CIN activates the cGAS‐STING‐NF‐κB pathway to produce inflammatory cytokines, predominantly including IL6 and IL8. IL6 promotes the survival of carcinoma cells with high CIN and thus promotes metastasis.^[^ ^21^ ^]^ However, the effects of mechanical force‐induced cGAS activation in carcinoma cells are still unclear.

Plenty of studies have shown that cGAS may regulate the secretion of chemokines, such as IL6, TNFα, CCL2, and CXCL1, ^[^ ^29^, ^30^, ^31^ ^]^ through cGAS‐STING pathway activation, which in turn promotes inflammatory prometastatic niche formation and recruits more immunocytes infiltration. It is well known that the recruitment and retention of monocytes to metastatic sites are primarily regulated by the CCL2‐CCR2 axis,^[^ ^32^, ^33^, ^34^ ^]^ where they differentiate into macrophages and promote cancer progression. On the other hand, some recent work has also revealed the noncanonical functions of cGAS in subcellular compartments other than the cytosol. For instance, nuclear cGAS anchors to chromatin and suppresses homologous recombination‐mediated DNA repair in response to genotoxic stress‐induced DNA damage,^[^ ^35^, ^36^ ^]^ thereby directly promoting cancer cell proliferation independently of STING. Nuclear cGAS also interacts with replication fork proteins in a DNA binding‐dependent manner and slows replication forks, which ultimately slows cell proliferation. ^[^ ^37^ ^]^ cGAS localized to mitochondria can protect hepatocellular carcinoma cells from ferroptosis, thus promoting cancer progression. ^[^ ^38^ ^]^ Nevertheless, whether mechanical force‐induced cGAS activation and chemokine secretion play a crucial role in pro‐metastatic niche formation, is entirely unknown.

In carcinoma cells, chemokine secretion requires the involvement of inflammatory cells, and whether splenocytes are involved in this process is unclear. The spleen and liver are connected anatomically and physiologically. All splenic blood perfuses to the liver through the HPV. The spleen has already been proven to serve as a monocyte reservoir to accommodate the demands of rapid‐onset inflammation in conditions such as ischemic myocardial injury, ischemic brain injury, atherosclerotic lesions, and hepatopulmonary syndrome.^[^ ^39^, ^40^, ^41^, ^42^, ^43^ ^]^ Our recent findings provide direct evidence that splenic monocytes migrate into the liver and shift to macrophages in mouse liver fibrosis progress.^[^ ^44^ ^]^ Macrophages are the most abundant immune cells in the liver, and the recruitment of monocyte‐derived macrophages is the major player during liver inflammation.^[^ ^45^, ^46^, ^47^, ^48^ ^]^ Clinical correlative data and a plethora of preclinical studies in mouse models of cancers have shown that tumor‐associated macrophages (TAMs) promote cancer development.^[^ ^47^, ^49^, ^50^, ^51^ ^]^ Therefore, the spleen might play a crucial role in the formation of pro‐metastatic inflammatory niche in the liver by exporting splenocytes under metastatic conditions.

Therefore, we hypothesize that in liver metastasis, carcinoma cells pass through the capillaries leading to nuclear deformation and cGAS activation. The latter mediates the inflammatory cytokine production and the infiltration of splenocytes, which facilitates liver metastasis. In this study, we used fluorescence and intravital liver imaging in a via hepatic portal vein (VHPV) metastasis of mouse and cell compression in vitro to observe the nuclear deformation and cGAS activation. ^[^ ^52^ ^]^ Manipulating cGAS activation and expression by inhibitor and knockout technology, we verify the effects of the cGAS pathway on liver metastasis in vitro and in vivo. Finally, we analyze the effects of splenocytes on liver metastasis under the cGAS activation. We conclude that mechanical force induces cGAS activation in carcinoma cell to recruit monocytes/macrophages to facilitate liver metastasis. The findings reveal a mechanism and provide a practical application for controlling liver metastasis.

## 2 Results

### 2.1 Nuclear Deformation and cGAS Activation Occur in VHPV Metastasis by Intravital Imaging

**Figure 1:**
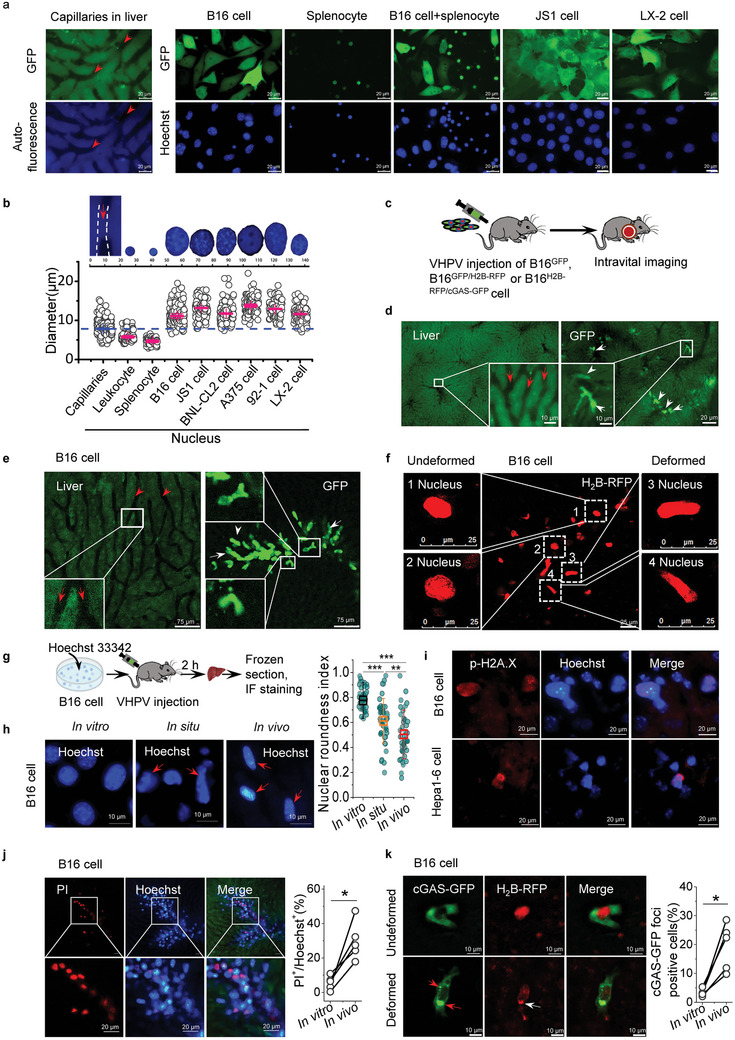
Nuclear deformation and cGAS activation occur in VHPV metastasis by intravital imaging. a) Representative fluorescence images of B16, JS1 and LX‐2 cells stably expressing GFP, splenocyte and leukocyte stably expressing GFP isolated from GFP transgenic mice, and intravital images of liver capillaries in GFP mice. Nuclei were stained with Hoechst 33342. Scale bars, 20 µm. b) Quantification of liver capillary diameter and nuclear diameter of multiple immune cells, carcinoma cells and hepatic cells. Data represent the mean ± s.d. of 3 independent experiments. c) Experimental schema for intravital imaging of metastasis experiments. VHPV, via hepatic portal vein. d) Representative intravital images of deformed B16^GFP^ cell in liver capillaries after portal vein injection to C57BL/6 mice (4 h after injection). Scale bars, 20 µm. e) Representative confocal intravital images of deformed B16^GFP^ cell in liver capillaries of C57BL/6 mice (4 h after injection). Scale bars, 75 µm. f) Representative confocal intravital images of B16 cells stably expressing H2B‐RFP in liver capillaries of C57BL/6 mice after VHPV injection. Images showing deformed and undeformed nuclei of B16^H2B‐RFP^ cells in liver capillaries. Scale bars, 25 µm. g) Experimental schema for detecting the morphology of carcinoma cell nucleus and DNA damage on liver section by immunofluorescence (IF) staining. h) Representative fluorescence images of in vitro, in situ and in vivo of B16 cells showing the deformed nuclei and the data showing nuclear roundness index. Red arrowheads point to deformed nuclei. Scale bars, 10 µm. i) Immunofluorescence histochemistry of in situ carcinoma cells in liver showing p‐H2A.X‐positive nuclei. Hoechst‐labeled cells are carcinoma cells injected VHPV in mouse liver. Scale bars, 20 µm. j) Representative fluorescence intravital images of B16 cell nuclei in liver capillaries of C57BL/6 mice. Data showing NE rupture in liver capillaries. Graphical quantification showing the percentage of ruptured nuclei (PI^+^) of B16 cells in mouse livers and in culture (*n* = 5 mice). C57BL/6 mice were inoculated with B16 cells stained with Hoechst 33342 (DNA, blue) and PI (propidium iodide, Red) via HPV injection and intravital imaged the liver in 30 min. Scale bars, 20 µm. k) Representative confocal intravital images of B16^H2B‐RFP^ cells expressing cGAS‐GFP in liver capillaries of C57BL/6 mice. Data showing cGAS activation in the liver. Graphical quantification showing the percentage of cGAS‐GFP foci formed B16 cells in mouse livers and in vitro (*n* = 5 mice). Scale bars, 10 µm. Red arrowheads point to cGAS‐GFP foci formation in the liver. Two‐tailed unpaired Student's t‐test was performed for the statistical significance. *, **, and *** stand for *p* < 0.05, <0.01, and <0.001, respectively. The images in (d)–(k) have been modified to improve brightness and contrast.

The nuclei of carcinoma cells, like B16, 92‐1, and A375, are much larger than the lumen dimensions of capillaries in the liver (11.1–13.7 µm versus 7.8 µm), the nuclei of other hepatic cell lines like JS1, LX‐2 and BNL‐CL2 are similar in size to carcinoma cells (11.6–13.1 µm versus 11.1–13.7 µm). However, the nuclei of immune cells are smaller than the lumen dimensions of capillaries in liver (4.6–5.7 µm versus 7.8 µm), which allows immune cells to pass through the capillaries unimpeded (**Figure** **1**a,b, Figure S1a, Supporting Information). To explore what happens when carcinoma cells move through capillaries, we engineered B16 cells to express GFP protein (B16^GFP^ cells, also used LX‐2^GFP^ and JS1^GFP^ cells) and inoculated these cells in the VHPV metastasis model. Based on the advancement of intravital imaging, B16^GFP^ cells in liver capillaries were visualized at single‐cell resolution through an abdominal window (Figure 1c). The GFP‐positive cells were stuck in the capillaries within minutes of arrival and the cytoplasm began to deform within hours (Figure 1d,e, Figure S1b, Supporting Information). Furthermore, nuclear deformation of these cells was also observed by using B16^GFP^ cells expressing H_2_B‐RFP in the nucleus (B16^GFP/H2B‐RFP^, also used LX‐2^GFP/H2B‐RFP^ and JS1^GFP/H2B‐RFP^ cells), and these deformed nuclei were elongated to fit the shape of liver capillaries (Figure 1f, Figure S1c–f, Supporting Information). The nuclear roundness of Hoechst‐labelled carcinoma cells was also measured in VHPV metastatic mouse liver by intravital imaging and in frozen liver sections by fluorescent imaging, the carcinoma cell in capillaries displayed a larger number of deformed and elongated nuclei (Figure 1g,h). We further used a combination of lamin A/C and DAPI or Hoechst staining and measured for nuclear roundness, we were able to find several of such deformed nuclei of Hoechst‐labelled carcinoma cells in the liver capillaries (Figure S1g, Supporting Information). In addition, nuclei positive for H2A.X(S139) were also detected in these Hoechst‐labelled carcinoma cells in liver section (Figure 1i). These suggest that the carcinoma cells in liver capillaries exhibit pronounced nuclear deformation associated with elevated DNA damage.

We also asked whether NE rupture can be detected in liver capillaries. The Hoechst‐labeled B16 cells (also used MHCC‐97H and BEL7404 cells) added with PI (propidium iodide) were inoculated to VHPV metastasis model, a higher percentage of PI‐positive nuclei were observed in the liver capillaries compared with control cells in vitro (Figure 1j, Figure S1h, Supporting Information). Since PI cannot stain nuclei with intact NE, these results suggest that NE rupture occurs in vivo. The nuclear envelope encases the genetic material, and NE rupture results in DNA leakage to cytosol. Thus, we engineered the B16^H2B‐RFP^ cells to express the fusion protein cGAS‐GFP (B16^cGAS‐GFP/H2B‐RFP^, also used Hepa1‐6^cGAS‐GFP^ cell) and inoculated these cells to VHPV metastasis model. By using intravital imaging, cGAS‐GFP foci around the RFP^+^/Hoechst^+^ nuclei were observed in liver capillaries, while cGAS‐GFP distributed dispersive in the cytoplasm of undamaged cells (Figure 1k, Figure S1i, Supporting Information). Furthermore, compared to control cells in vitro, carcinoma cells exhibited a higher proportion of cGAS‐GFP foci‐positive signals in liver capillaries (Figure 1k). These data demonstrate that the mechanical force exerted on the carcinoma cells can dynamically deform the nuclei and result in NE rupture, which induces cGAS activation by cytoplasmic DNA in the liver capillaries.

### 2.2 Nuclear Deformation and cGAS Activation are Induced by Cell Compression in Vitro and then Exacerbate VHPV Metastasis in Liver

**Figure 2:**
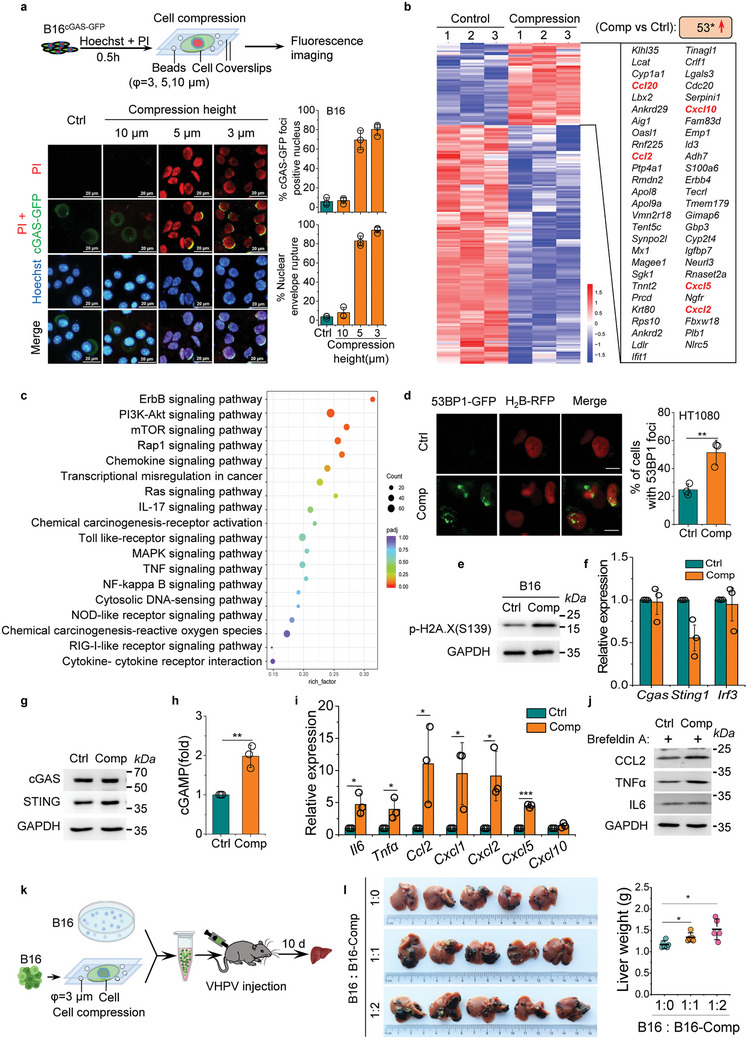
Nuclear deformation and cGAS activation are induced by cell compression in vitro. a) Experimental schema of cell compression experiments and representative fluorescence images of B16 cell nuclei under compression. B16 cells expressing cGAS‐GFP (Green) were stained with Hoechst (DNA, blue) and PI (DNA, Red) and compressed at different heights (3,5,10 µm). PI‐stained nuclei (Red) indicated NE rupture under cell compression, and perinuclear dotted or crescent‐shaped cGAS‐GFP foci indicated cytoplasmic DNA leakage. The images were acquired in 20 min after cell compression. Quantification of cGAS foci formatted cells and NE ruptured cells during cell compression is shown. Scale bars, 20µm. b) Heatmaps of RNA‐seq data depicted the genes up‐regulated by cell compression treatment in B16 cells compared with their untreated counterparts. c) KEGG pathway enrichment analysis was performed using the upregulated genes. d) Representative fluorescence images of DNA damage in HT1080 cells stably expressing 53BP1‐GFP and histone H_2_B‐RFP under compressed. Graphical quantification showing 53BP1‐foci‐positive cells in control and compressed cells. Scale bars, 10 µm. e) Western blotting analysis showed the levels of phospho‐H2A.X(S139) in cell lysates from control or compressed B16 cells. GAPDH is the loading control. f) Real time‐PCR analysis of cGAS‐STING‐IRF 3 pathway genes at 24 h under control and compressed cells. g) Western blotting analysis showed the levels of cGAS, STING and IRF 3 in cell lysates from control or compressed B16 cells at 24 h after treatment. GAPDH is the loading control. h) Detection of cGAMP by ELISA in cell lysates from control or compressed B16 cells. i) Real time‐PCR analysis of inflammatory and cytokine genes expression in B16 cells under control or cell compression at 24 h. j) Western blotting analysis of inflammatory and cytokine genes expression under control or cell compression at 24 h. GAPDH is the loading control. Brefeldin A is used to block protein transport. k) Experimental schema for cell compression and mixed carcinoma cell inoculation in VHPV mice model. l) Representative images showing metastatic tumor burden on the liver surface of mice inoculated with B16 cells mixed with high compression treatment B16 cells at indicated ratio. Graphical quantification showing the weight of liver (mean ± s.d., *n* = 5 mice). Hoechst, Hoechst33342; PI, propidium iodide; Ctrl, control; Comp, Compressed at 3 µm height. Data are representative of three (a–j) independent experiments. Mean ± s.d., two‐tailed unpaired Student's *t*‐test were performed for the statistical significance. *, **, and *** stand for *p* < 0.05, <0.01, and <0.001, respectively.

To further confirm the cGAS activation induced by mechanical force, we introduced cell compression by covering cells with two nonadhesive surfaces in vitro. Interphase carcinoma cell nuclei range from 11.1 to13.7 µm in diameter, thus the cell compression heights were limited to 10, 5, and 3 µm. The B16^cGAS‐GFP^ cells were incubated with Hoechst and PI, then mixed these cells with glass beads (diameter: 3, 5, and 10 µm,) and compressed to indicated heights between two nonadhesive surfaces (**Figure** **2**a). In low compression group (10 µm), the nuclei were slightly deformed and the NE was intact. In middle compression group (5 µm), the nuclei deformed and began to form bubbles around the nuclei, NE rupture occurred, and a crescent‐shaped cGAS‐GFP foci formed around the nucleus. In high compression group (3 µm), the proportion of nuclei with NE rupture exceeded 94%, and cGAS‐GFP foci positive cell reached 80% (Figure 2a). NE rupture was observed in multiple cell lines at high compression (Figure S2a–c, Supporting Information). Similarly, cGAS‐GFP foci were also detected when B16^cGAS‐GFP^ cells crossed a pore size of 5 µm in the transwell membrane (Figure S2d, Supporting Information), which implied NE rupture and DNA leakage occurring under confinement microenvironment. Therefore, we chose to limit subsequent investigation to 3 µm height compression.

Furthermore, we performed RNA sequencing (RNA‐seq) on cell compression or untreated B16 cells. We analyzed the transcriptionally activated genes response to mechanical force treatment, 53 such upregulated genes were uncovered in compressed B16 cells compared with their untreated counterparts (Figure 2b). Five of these upregulated genes were chemokines, including *Ccl2, Ccl20, Cxcl2, Cxcl5* and *Cxcl10* (Figure 2b). Approximately half of these genes (23 of 53 genes) were reversed by *Cgas* deletion, specifically including these five chemokines (Figure S2e, Supporting Information). The Kyoto Encyclopedia of Genes and Genomes (KEGG) analysis further highlighted the role of cGAS in response to mechanical force. When comparing the upregulated differentially expressed genes of carcinoma cells between control and compressed B16 cells, we observed enrichment in pathways including cytosolic DNA‐sensing pathway, together with chemokine, NF‐κB, TNF, and TGF‐β signaling pathways (Figure 2c). As expected, high compression also increased 53BP1‐GFP foci formation in HT1080^53BP1‐GFP/H2B‐RFP^ cells (Figure 2d), and led to an elevated phospho‐H2A.X(S139) in B16 cells (Figure 2e). These experiments indicated the existence of damaged DNA and cGAS pathway activation in the compressed cells. To further validate mechanical force‐induced activation of the cGAS pathway, we detected components of cGAS pathway in carcinoma cells under high compression. Cell compression did not change the expression of *Cgas, Sting1*, and *Irf3* (Figure 2f,g), but the amount of cGAMP (a product of cGAS enzyme) increased in compressed cells (Figure 2h). And the downstream genes of cGAS pathway, such as proinflammatory factors *Il6, Tnfa*, and the chemokines *Ccl2*, *Cxcl1, Cxcl2, Cxcl5* were upregulated in compressed cells (Figure 2i,j, Figure S2f, Supporting Information). Together, our data indicate that the increased mechanical force results in NE rupture, DNA damage and DNA leakage, which in turn triggers cGAS activation and enhances the transcriptional activity of cGAS‐dependent chemokines.

To investigate the effect of cell compression on liver metastasis, we mixed B16 cells with high compression treated B16 cells at various ratio and inoculated these mixed cells VHPV to metastatic mouse model (Figure 2k). Interestingly, compared to inoculating untreated B16 cells, high compression treatment carcinoma cells exacerbated liver metastasis (Figure 2l). These data provide evidence for the mechanical force exerting on carcinoma cell promotes liver metastasis in vivo.

### 2.3 Nuclear Deformation and cGAS Activation of Carcinoma Cell Enhances the Migration of Macrophages in Vitro

**Figure 3:**
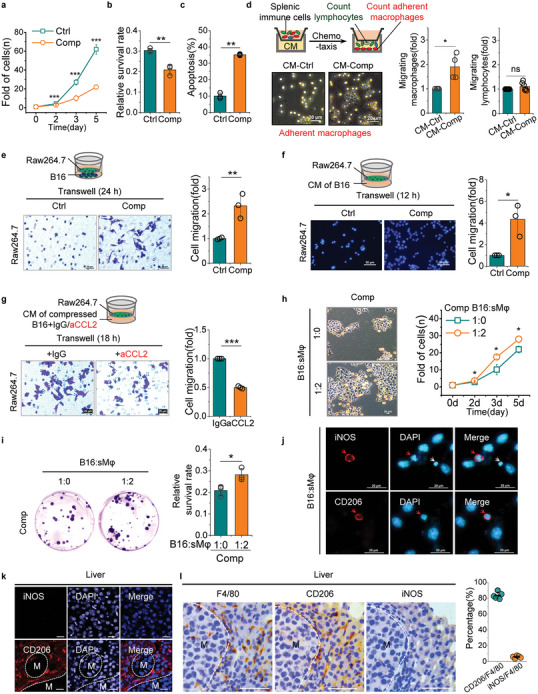
Nuclear deformation and cGAS activation of carcinoma cell enhances the migration of macrophages in vitro. a) Proliferation rate of control and compressed B16. b) Colony formation of control and compressed B16 cells. c) Apoptotic cell fraction quantified by Annexin V staining of control and compressed B16 cells at 48 h. d) Schematic of transwell migration assay of splenic immune cells in the presence of CM collected from control and compressed B16 cells. Representative images and quantitation of adherent macrophages in the bottom transwell chamber, and quantitation of suspension cells (lymphocytes) in the bottom transwell chamber. Scale bar, 20 µm. e) Migration of Raw264.7 cells recruited by control and compressed B16 cells. Scale bar, 50 µm. f) Migration of Raw264.7 cells recruited by CM of control and compressed B16 cells. Scale bar, 50 µm. g) Transwell migration assay of Raw264.7 cells by CM of compressed B16 cells in the presence of CCL2‐neutralizing or IgG antibodies. Scale bar, 20 µm. h) Proliferation rate of compressed B16 cells were determined by coculturing with splenic macrophages at the indicated ratio. Scale bar, 20 µm. i) Clonogenic assay to detect colony formation of compressed B16 cells co‐culture with splenic macrophages at indicated ratio. j) Representative immunofluorescence images of M1 and M2 polarized macrophages when splenic macrophages were cocultured with B16 cells for 48 h. Scale bar, 20 µm. k) Representative immunofluorescence images of iNOS^+^(M1) and CD206^+^(M2) macrophages in paraffin‐embedded liver tissue of VHPV metastasis mouse model. Scale bars, 20µm. M, metastatic foci. l) Representative histological images of iNOS^+^ and CD206^+^ macrophages staining in serial sections of paraffin‐embedded liver tissue of VHPV metastasis mouse model, and quantification of M1(iNOS^+^) and M2 (CD206^+^) type macrophages. Scale bars, 20µm. (Mean ± s.d., *n* = 5 mice). Ctrl, control; Comp, Compression at 3 µm height; sMφs, splenic microphages; CM, culture medium. Data showing in (a–i) are representative of more than three independent experiments. Mean ± s.d., two‐tailed unpaired Student's t‐test were performed for the statistical significance. *, **, and *** stand for *p* < 0.05, <0.01, and <0.001, respectively.

To assess the effect of mechanical force on carcinoma cells, we detected the ability of cell proliferation, colony formation and apoptosis in compressed B16 cells. As expected, the mechanical force exerting on carcinoma cell suppressed cell proliferation, decreased cell survival and resulted in apoptosis (**Figure** **3**a–c). Considering that cell compression induced cGAS activation and resulted in chemokines expression (Figure 2i,j, Figure S2f, Supporting Information), the damaged carcinoma cells may remodel the inflammatory niche and thus alter the fate of these cells. To illuminate this issue, we assessed the ability of damaged carcinoma cells to recruit spleen immune cells in the transwell system. Conditioned medium (CM) from damaged carcinoma cells promoted transwell migration of macrophages, but the CM did not affect lymphocytes migration in this assay (Figure 3d). By using splenic macrophages and cell line of macrophage, we further confirmed that cell compression greatly enhanced the ability of carcinoma cells to recruit macrophages (Figure 3e, Figure S3a, Supporting Information), the soluble factors secreted by compressed carcinoma cells was responsible for recruiting macrophages (Figure 3f). Furthermore, blocking the CCL2 in CM from compressed carcinoma cells by neutralizing antibody could abolish macrophages migration (Figure 3g). In our coculture experiments, the data suggest that it is macrophage, rather than lymphocyte, that improve the proliferation and survival of compressed carcinoma cells (Figures 3h,i, Figures S3b–d, Supporting Information). Macrophages contact with cancer cells in vivo can develop to TAMs. TAMs are suppressive to the activities of cytotoxic T and natural killer cells that have the potential to eradicate tumors, thus it is general consensus that TAMs promote tumor progression and metastasis. We further detected the polarity of macrophages in co‐culture experiments and in liver sections of VHPV metastasis mouse model by immunofluorescent staining of the arginine metabolism marker iNOS (inducible nitric oxide synthase, expressed in M1 macrophages) or cell surface marker CD206 expressed on M2 macrophages. In co‐culture experiments, both M1 (iNOS^+^) and M2 (CD206^+^) type macrophages were present (Figure 3j). However, in liver sections of mouse model of VHPV metastasis, almost all macrophages were CD206 positive (Figure 3k,l), which was considered to be M2 type macrophages that support tumor growth. These data demonstrate that cGAS activation enhances the recruitment of macrophages, which promotes the proliferation and survival of these carcinoma cells in vitro and in vivo.

### 2.4 Nuclear Deformation of Carcinoma Cell Promotes Monocytes/Macrophages Infiltration in VHPV Metastasis

**Figure 4:**
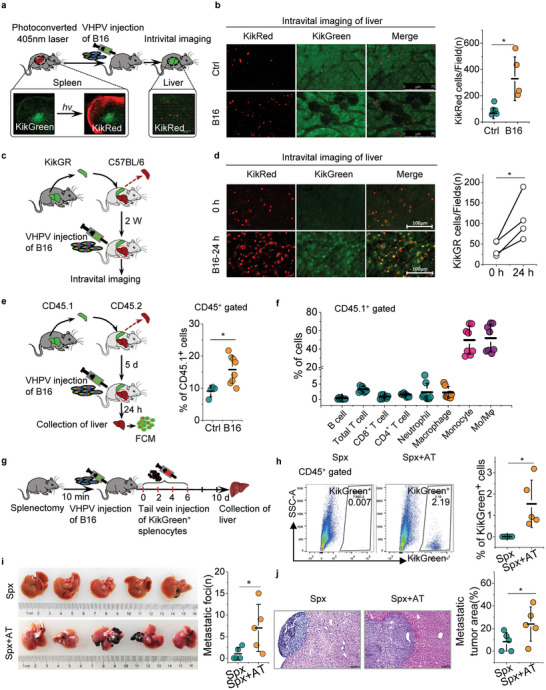
Nuclear deformation of carcinoma cells promotes splenocyte infiltration in VHPV metastasis. a) Surgical procedure for photoconversion experiments. b) Representative confocal intravital images of splenocytes (KikRed^+^) infiltration in liver capillaries of spleen‐specific photoconverted KikGR mice undergoing VHPV injection of PBS (ctrl) and B16 cells. Graphical quantification showing the count of KikRed^+^ splenocytes in livers. (Mean ± s.d., *n* = 4 or 5 mice) Scale bars, 75 µm. c) Experimental schema for spleen transplantation and liver intravital imaging experiment in the mouse model of VHPV metastasis. d) Representative confocal intravital images of liver splenocyte (KikRed^+^) infiltration in C57BL/6 mice undergoing spleen transplantation before (0 h) receiving VHPV injection with B16 cells and 24 h after receiving inoculation. Quantification graph shows the count of KikRed^+^ splenocyte infiltration in the liver before (0 h) and after the inoculation B16 cells. (*n* = 4 mice). Scale bar: 100µm. e) Experimental schema for spleen transplantation and flow cytometry assay of splenic immune cells in liver of VHPV mice model. Quantification showing the percentage of CD45.1^+^ splenocyte infiltration in liver of control(ctrl) and after B16 cells inoculated mice. f) Quantification showing the infiltrated spleen immune cell population in the livers of spleen transplant mice receiving VHPV injection of B16 cells (mean ± s.d., *n* = 8 mice). g) Scheme of splenocytes adoptive transfusion assays for (h–j). h) KikGreen splenocytes migrated to recipient liver were detected by flow cytometry after 10 days. Graphical quantification showing the count of KikGreen splenocytes in livers. i) Representative images showing the metastatic tumor burden on the surface of liver (black: metastatic melanoma foci), Graphical quantification showing the count of metastatic foci on the surface of livers. j) Representative images of hematoxylin and eosin (HE) stained liver sections showing tumor burden in livers. Graphical quantification showing the count of tumor area in HE‐stained liver sections. Scale bars, 100 µm. Spx, performed splenectomy; Spx+AT, performed splenectomy and adoptive transfusion of splenocytes. Mean ± s.d., *n* = 5 mice, two‐tailed unpaired Student's *t*‐test was performed for the statistical significance. * Stand for *p* < 0.05.

To interrogate the influence of mechanical force‐induced nuclear deformation and cGAS activation on the immunocytes infiltration in liver metastasis, we used photoconvertible KikGR transgenic mice to track the splenocytes in liver by intravital imaging in VHPV metastasis model (**Figure** **4**a). Photoconverted KikRed splenocytes were observed in control mouse liver, carcinoma cells invasion resulted in increased photoconverted KikRed splenocytes infiltration in liver (Figure 4b). In a second in vivo tracking experiments, the spleen transplanted C57BL/6 mice received spleen from KikGR mice donor were used (Figure 4c). Compared to mice without carcinoma cell inoculation, there were much more KikRed^+^ splenocytes infiltration in liver after B16 cells inoculation 24 h later (Figure 4d). Adoptive transfusion experiments also revealed that the carcinoma cells in liver capillaries exhibited increased splenocytes recruitment in vivo (Figure S4a,b, Supporting Information). In another spleen transplant experiment, the composition of the infiltrating spleen immune cell population in the liver was analyzed by using CD45.2 mouse receiving spleen from CD45.1 mouse donor (Figure 4e). Consistently, after inoculating B16 cells, the invading carcinoma cells recruited more CD45.1^+^ splenocyte infiltration in the liver (Figure 4e). Further analysis revealed that although splenic lymphocytes and neutrophils could also migrate to the liver, the liver invaded by carcinoma cells was significantly enriched with splenic monocytes/ macrophages (Figure 4f). These data provide evidence for the migration and infiltration of splenic monocytes/ macrophages in liver to manufacture of inflammatory niche.

To verify the role of splenocytes in liver metastasis, KikGreen^+^ CD11b^+^ splenocytes were transfused to C57BL/6 mice which performed splenectomy and VHPV inoculation of B16 cells (Figure 4g). Compared to the mice only performed splenectomy, mice received KikGreen^+^ CD11b^+^ splenocytes transfusion demonstrated Kikgreen^+^ splenocytes infiltration in liver (Figure 4h) and exacerbated liver metastasis (Figure 4i–j). These data demonstrate that nuclear deformation mediates splenocytes (mainly splenic monocytes/ macrophage) infiltration in liver and exacerbates liver metastasis.

### 2.5 cGAS‐STING Pathway Interrupting in Carcinoma Cell Attenuates Inflammatory Cell Infiltration and Ameliorates Liver Metastasis

**Figure 5:**
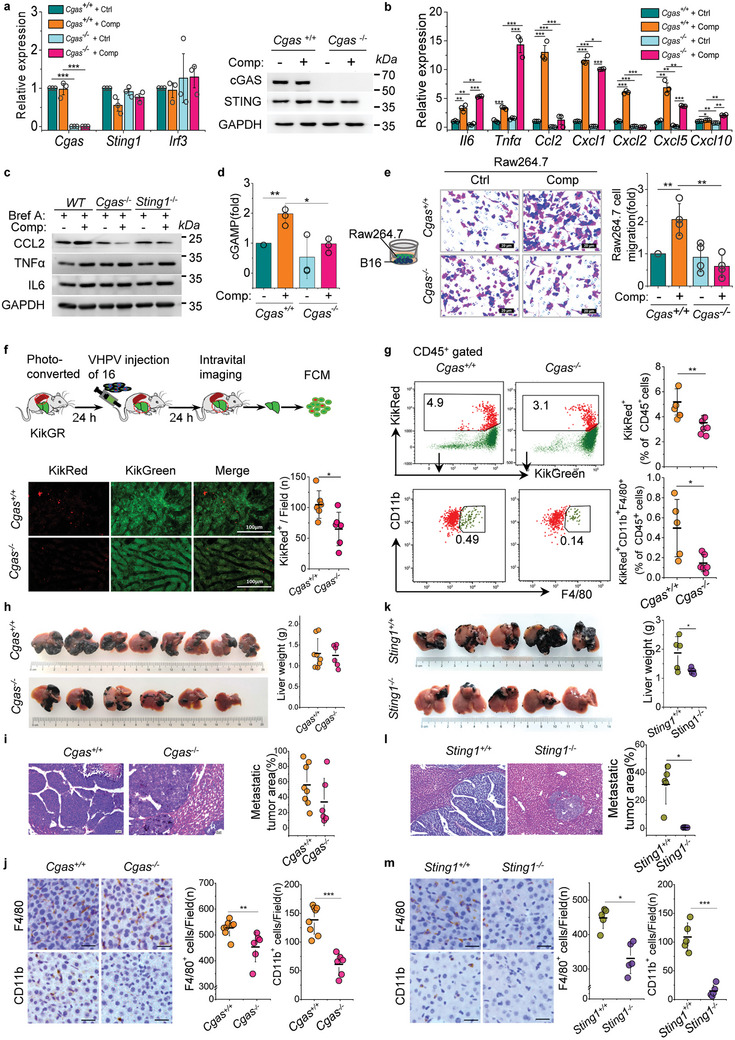
*Cgas* knockout in carcinoma cell attenuates inflammatory cell infiltration. a) qPCR and western blotting analysis of cGAS‐STING‐IRF3 pathway genes expression in control or compressed *Cgas^+/+^* and *Cgas^‐/‐^* B16 cells at 24 h. GAPDH is the loading control for western blotting. b) qPCR analysis of inflammatory and chemokine genes in control or compressed *Cgas^+/+^* and *Cgas^‐/‐^* B16 cells at 24 h. c) Western blotting analysis of inflammatory factors and chemokines in control and compressed wild‐type, *Cgas^‐/‐^* and *Sting1^‐/‐^* B16 cells at 24 h, GAPDH is the loading control. Brefeldin A (Bref A) is used to block protein transport. d) The cGAMP of control and compressed *Cgas^+/+^* and *Cgas^‐/‐^* B16 cells were measured by ELISA. e) Migration of Raw264.7 cells recruited by control or compressed *Cgas^+/+^* and *Cgas^‐/‐^* B16 cells. Scale bars, 20 µm. f) Experimental schema for photoconversion and intravital imaging experiments. Representative confocal intravital images of splenocyte (KikRed^+^) infiltration in the liver capillaries of spleen‐specific photoconverted KikGR mice undergoing VHPV injection of *Cgas^+/+^* and *Cgas^‐/‐^* B16 cells. Graphical quantification showing the count of KikRed^+^ splenocytes in the liver. (Mean ± s.d., *n* = 6 mice). Scale bars, 100 µm. g) The KikRed^+^ and KikRed^+^CD11b^+^F4/80^+^ population of splenocytes in liver were detected by FCM in (f). Graphical quantification showing the difference in KikRed^+^ and KikRed^+^CD11b^+^F4/80^+^ splenocyte infiltration in mouse livers inoculated with *Cgas^+/+^* and *Cgas^‐/‐^* B16 cells. (Mean ± s.d., *n* = 5 or 6 mice). h) Representative images showing metastatic tumor burden on the liver surface of mice inoculated with *Cgas^+/+^* and *Cgas^‐/‐^* B16 cells VHPV. Graphical quantification showing the weight of livers. i) Representative images showing tumor area in HE‐stained liver sections of (h). Graphical quantification showing the metastatic tumor area of HE‐stained liver sections. Scale bars, 20 µm. j) Representative micrographs of liver sections from *Cgas^+/+^* and *Cgas^‐/‐^* B16 cells inoculated mice stained with the myeloid cell markers CD11b and F4/80, and representative quantification of the count of CD11b^+^ cells and F4/80^+^ cells per field. Scale bars, 20 µm. (Mean ± s.d., *n* = 6 or 8 mice). k) Representative images showing metastatic tumor burden on the liver surface of mice inoculated with *Sting1^+/+^* and *Sting1^‐/‐^* B16 cells VHPV. Graphical quantification showing the weight of livers. l) Representative images showing tumor area in HE‐stained liver sections of (k). Graphical quantification showing the metastatic tumor area of HE‐stained liver sections. Scale bars, 20 µm. m) Representative micrographs of liver sections from *Sting1^+/+^* and *Sting1^‐/‐^* B16 cells inoculated mice stained with CD11b and F4/80, and representative quantification of the count of CD11b^+^ cells and F4/80^+^ cells per field. Scale bars, 20 µm. (Mean ± s.d., *n* = 5 mice). Data are representative of three (a‐b, d) or four (c, e) independent experiments. Mean ± s.d., two‐tailed unpaired Student's *t*‐test were performed for the statistical significance. *, **, and *** stand for *p* < 0.05, <0.01, and <0.001, respectively. FCM, flow cytometry.

cGAS activation is crucial for mechanical force‐induced proinflammatory factors and chemokines expression. Of note, *Cgas* knockout did not alter the expression of *Sting1* and *Irf3* (**Figures** **5**a and S5a, Supporting Information), but *Cgas* deletion almost completely abolished cell compression induced cGAMP production and chemokine CCL2, CXCL2 expression, and slightly attenuated CXCL5 expression (Figure 5b–d; Figure S5c, Supporting Information). *Sting1* deletion also reversed cell compression induced CCL2 expression (Figure 5c; Figure S5d, Supporting Information). Consistent with literature reported, *Cgas* deletion accelerated cell proliferation and enhanced cell survival (Figure S5b,f, Supporting Information), ^[^ ^37^ ^]^ while *Sting1* deletion only diminished cell survival but did not affect cell proliferation (Figure S5e,f, Supporting Information). In addition, when cGAS and STING were deficient, the chemotactic migration of macrophages by mechanic compressed carcinoma cells was impaired (Figure 5e; Figure S5g, Supporting Information). To clarify the function of cGAS in recruiting splenocytes into the metastatic niche in vivo, we used KikGR mice combined with liver intravital imaging to track splenocytes migration in VHPV metastasis model (Figure 5f). Importantly, less photoconverted KikRed splenocyte infiltration was observed in mice inoculated with *Cgas^‐/‐^* B16 cells compared to mice inoculated with *Cgas^+/+^* B16 cells (Figure 5f). Flow cytometry analysis further confirmed that both KikRed^+^ and KikRed^+^CD11b^+^F4/80^+^ splenocyte populations in liver of mice inoculated with *Cgas^‐/‐^* B16 cells was less than those inoculated with *Cgas^+/+^* B16 cells (Figure 5g). In cell transfusion and tracking experiments, it was also confirmed that fewer splenic CD45^+^KikRed^+^ cells were recruited by *Cgas^‐/‐^* B16 cells to liver than *Cgas^+/+^* B16 cells (Figure S5h,I, Supporting Information). Consistent with these results, transfusion of CD45.1^+^CD11b^+^ splenocytes into CD45.2 mice inoculated with *Cgas^+/+^* or *Cgas^‐/‐^* B16 cells in VHPV metastasis model also demonstrated that *Cgas* knockout in carcinoma cells impaired splenocyte recruitment to liver (Figure S5j,k, Supporting Information). These data confirm that cGAS pathway deficiency ameliorates inflammatory cell infiltration in liver metastasis.

However, *Cgas* knockout did not ameliorate liver metastasis in VHPV mice model (Figure 5h,i), but *Sting1* knockout significantly ameliorated liver metastasis (Figure 5k,l). Of note, compared to mice inoculated with wild type B16 cells, both *Cgas* and *Sting1* knockout in carcinoma cells mitigated the CD11b^+^ and F4/80^+^ myeloid cells infiltration in liver (Figure 5j,m). As expected, although cGAS‐STING pathway blockage attenuated myeloid cell infiltration in the liver, macrophages presented in the metastatic niche were predominantly M2 polarized (Figure S5l, Supporting Information). Taken together, interruption of the cGAS‐STING pathway attenuates inflammatory cell infiltration in liver metastatic niche, but only *Sting1* knockout ameliorates liver metastasis. This suggests that cGAS plays a complex role in liver metastasis.

### 2.6 Inhibition of cGAS Activity Attenuates Inflammatory Cell Infiltration and Ameliorates Liver Metastasis

**Figure 6:**
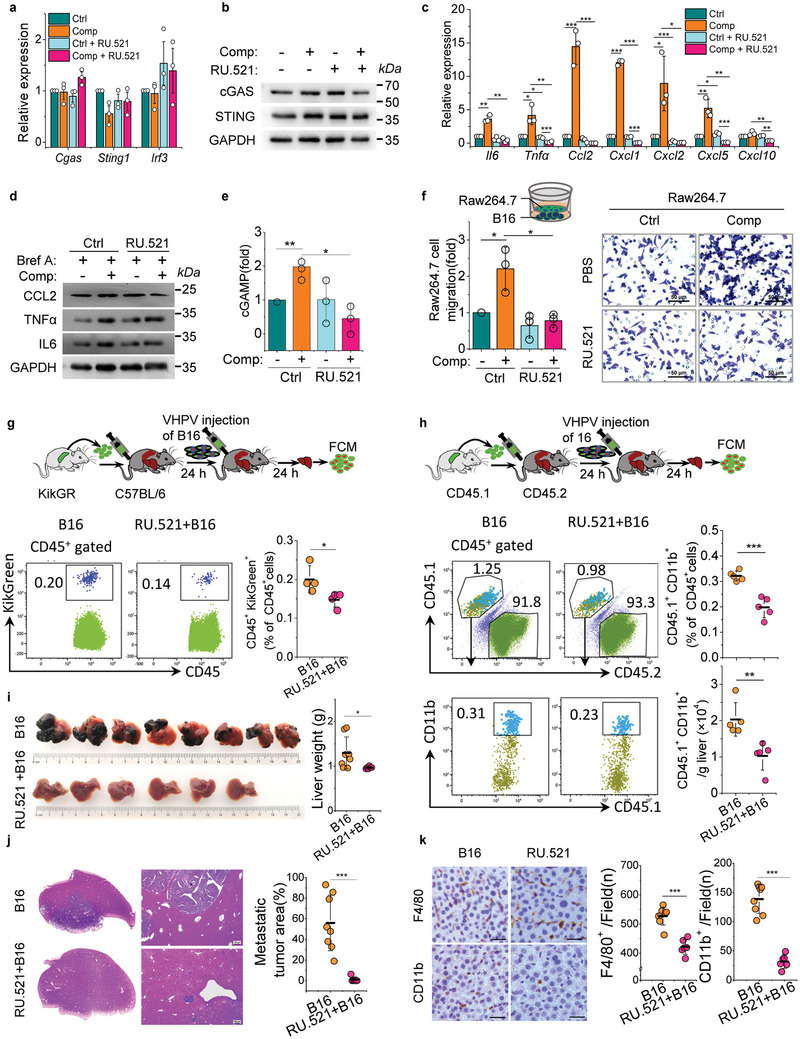
Inhibition of cGAS activity attenuates inflammatory cell infiltration and ameliorates liver metastasis. a) qPCR analysis of cGAS‐STING‐IRF3 pathway genes expression in control and compressed B16 cells in the presence or absence of RU.521. b) Western blotting analysis of cGAS‐STING‐IRF3 pathway genes expression in control and compressed B16 cells in the presence or absence of RU.521. GAPDH is the loading control. c) qPCR analysis of inflammatory and chemokine genes in control and compressed B16 cells at 24 h in the presence or absence of RU.521. d) Western blotting analysis of inflammatory factors and chemokines at 24 h under control or compressed B16 cells in the presence or absence of RU.521, GAPDH is the loading control. Brefeldin A (Bref A) was used to block protein transport. e) The cGAMP of control and compressed B16 cells in the presence or absence of RU.521 were measured by ELISA. f) Migration of Raw264.7 cells recruited by control and compressed B16 cells in the presence or absence of RU.521. Scale bars, 50 µm. g) Scheme of splenocyte adoptive transfusion assays. The CD45^+^KikGreen^+^ splenocyte infiltration in the liver were detected by FCM in mice inoculated with B16 cells treatment with or without RU.521. Graphical quantification showing the difference of CD45^+^KikGreen^+^ splenocyte infiltration in mouse liver inoculated with B16 cells treatment with or without RU. 521. (Mean ± s.d., *n* = 4 mice). h) The CD45.1^+^ CD11b^+^ splenocyte was detected by FCM in mice inoculated with B16 cells treatment with or without RU.521. Graphical quantification showing the difference of CD45.1^+^ CD11b^+^ splenocyte infiltration in mouse liver inoculated with B16 cells treatment with or without RU.521. (Mean ± s.d., *n* = 5 mice). i) Representative images showing metastatic tumor burden on the liver surface of mice inoculated with B16 cells treated with or without of RU.521. Graphical quantification showing the weight of livers. (Mean ± s.d., *n* = 6 or 8 mice). j) Representative images showing tumor area in HE‐stained liver sections of (i). Graphical quantification showing the metastatic tumor area of HE‐stained liver sections. k) Representative micrographs of liver sections from B16 cells and RU.521 treatment B16 cells inoculated mice stained with CD11b and F4/80, and representative quantification of the count of CD11b^+^ cells and F4/80^+^ cells per field. Scale bars, 20 µm. Data are representative of three (a–f) independent experiments. Mean ± s.d., two‐tailed unpaired Student's *t*‐test were performed for the statistical significance. *, **, and *** stand for *p* < 0.05, <0.01, and <0.001, respectively. FCM, flow cytometry.

*Cgas* knockout expedited carcinoma cell proliferation and enhanced cell survival in vitro. This indicated the pleiotropy of the cGAS. To determine the role of cGAS in liver metastasis, the cGAS inhibitor RU.521 was used to block enzyme activity. cGAS inhibitor treatment barely interfered with the expression of cGAS, STING and *Irf3* (**Figure** **6**a,b), but impaired the expression of inflammatory factors IL6, TNFα and chemokines like CCL2, CXCL1, CXCL2, and CXCL5 induced by cell compression (Figure 6c,d). cGAS inhibitor also hindered the production of cell compression induced cGAMP (Figure 6e). Furthermore, the recruitment of Raw264.7 cells by compressed carcinoma cells was assessed in the transwell system. As expected, the chemotactic migration of Raw264.7 cells by mechanical compressed carcinoma cells was impaired by cGAS inhibitor (Figure 6f). By analyzing migration using conditioned media, it was demonstrated that cGAS inhibitor treatment blocked chemokine expression in compressed carcinoma cells, ultimately blocking macrophage migration (Figure S6a, Supporting Information).

To obtain in vivo evidence for cGAS inhibitor function, splenic KikGreen^+^CD11b^+^ splenocytes were transfused into C57BL/6 mice, and then B16 cells and RU.521 treated B16 cells were inoculated into mouse livers VHPV, respectively (Figure 6g). Compared to mice inoculated with B16 cells, mice inoculated with cGAS inhibitor treated B16 cells showed less CD45^+^KikGreen^+^ splenocyte infiltration in liver (Figure 6g). Furthermore, in a second adoptive transfer mice model, less CD45.1^+^CD11b^+^ splenocyte infiltration was observed in CD45.2 mice inoculated with cGAS inhibitor treated B16 cells compared to mice inoculated with B16 cells (Figure 6h). These results suggest that cGAS enzyme activity in carcinoma cell is crucial for inflammatory cell infiltration in liver metastasis.

cGAS inhibitor decreased cell survival but did not influence cell proliferation in vitro (Figure S6b–c, Supporting Information). To further explore the function of cGAS inhibitor in liver metastasis, B16 cells and cGAS inhibitor treated B16 cells were inoculated to mouse livers VHPV. Importantly, cGAS inhibitor treatment ameliorated liver metastasis (Figure 6i,j). Consistently, compared to mice inoculated with B16 cell, mice inoculated with cGAS inhibitor treated B16 cell showed less amounts of CD11b^+^ and F4/80^+^ myeloid cells infiltration in livers, and most of the F4/80^+^ macrophages were M2‐polarized (Figure 6k, Figure S6d, Supporting Information). Taken together, these data provide evidence for cGAS enzyme activity inhibition in carcinoma cells attenuate inflammatory cell infiltration and thus ameliorates liver metastasis.

### 2.7 Both cGAS Activation and Splenocyte Infiltration are Crucial for Liver Metastasis

**Figure 7:**
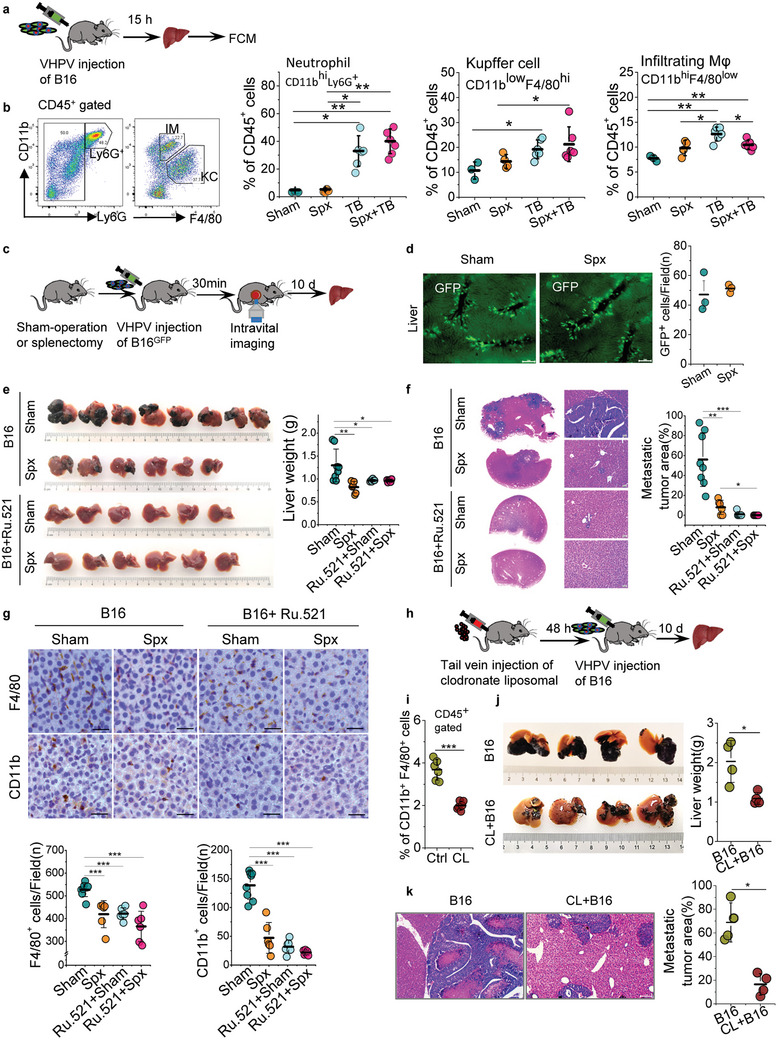
Both cGAS activation and splenocyte infiltration are crucial for liver metastasis. a) The experimental scheme to investigate the function of the spleen in myeloid cell infiltration in liver metastatic niche. b) Representative cytometry for gating neutrophils (CD45^+^CD11b^hi^Ly6G^+^), Kupffer cells (CD45^+^Ly6G^‐^CD11b^low^ F4/80^hi^) and infiltrating macrophages (CD45^+^Ly6G^‐^CD11b^hi^ F4/80^low)^ of liver from mice inoculated with B16 cells and PBS after performed sham‐operation or splenectomy. Graphical quantification showing the frequencies of these cells in liver. (Mean ± s.d., *n* = 3, 4 or 6 mice). c) Experimental scheme of intravital imaging experiments in VHPV metastasis model. d) Representative intravital images showing the distribution of B16^GFP^ cells in the liver capillaries after VHPV injection of B16^GFP^ cells in mice performed sham‐operation or splenectomy; Graphical quantification showing the B16^GFP^ cells in the liver capillaries per field. Scale bars, 100 µm. (Mean ± s.d., *n* = 3 mice). e) Representative images showing metastatic tumor burden on the liver surface of mice performed sham‐operation or splenectomy and inoculated with B16 cells treatment with or without of RU.521. Graphical quantification showing the weight of livers. (Mean ± s.d., *n* = 6 or 8 mice). f) Representative images showing tumor area in HE‐stained liver sections of (e). Graphical quantification showing the metastatic tumor area of HE‐stained liver sections. Scale bars, 25 µm. g) Representative micrographs of liver sections stained with the myeloid cell markers CD11b and F4/80, and representative quantification of the count of CD11b^+^ cells and F4/80^+^ cells per field. Scale bars, 20 µm. h) The experimental scheme to determine whether elimination of macrophages in spleen and liver affects the liver metastasis. i) Graphical quantification showing a marked reduction of macrophages (CD11b^+^F4/80^+^) in spleen of mice treated with Clodronate Liposomes (CL) compared to control mice treated with PBS Liposomes (Ctrl). (Mean ± s.d., *n* = 6 mice). j) Representative images showing liver surface tumor burden of mice treated with Clodronate Liposomes (CL) compared to control mice treated with PBS Liposomes after 10 days of B16 inoculation VHPV and graphical quantification showing liver weight. (Mean ± s.d., *n* = 4 mice). k) Representative images showing tumor area in HE‐stained liver sections of mice inoculated with B16 cells VHPV and injected with or without clodronate liposomal through tail vein. Graphical quantification showing the metastatic tumor area of HE‐stained liver sections. Spx, splenectomy; Sham, Sham‐operation; TB, Tumor bearing; Spx+TB, performed splenectomy and inoculated B16 cells VHPV; CL, clodronate liposomal. two‐tailed unpaired Student's *t*‐test were performed for the statistical significance. *, **, and *** stand for *p* < 0.05, <0.01, and <0.001, respectively.

To further explore the functional population of splenocyte in exacerbating liver metastasis, mice were performed sham‐operation or splenectomy, respectively, and then B16 cells were inoculated to mice VHPV (**Figures** **7**a and S7a, Supporting Information). Interestingly, splenectomy did not affect the myeloid cells distribution in mouse liver. However, carcinoma cell invasion markedly increased the myeloid cells infiltration in liver, including CD11b^hi^Ly6G^+^ neutrophils, CD11b^low^F4/80^hi^ Kupffer cells and CD11b^hi^F4/80^low^ monocyte‐derived infiltrating macrophages. Performed splenectomy before carcinoma cell inoculation reduced the monocyte‐derived infiltrating macrophages in liver, but did not affect the count of neutrophils and Kupffer cells (Figure 7b and Figure S7b, Supporting Information). These results suggest that spleen exporting monocytes/ macrophages are essential to accelerate the colonization of carcinoma cells in liver.

Furthermore, by using intravital imaging to detect B16^GFP^ cell distribution in mice performed sham‐operation or splenectomy in a VHPV metastasis model (Figure 7c), we confirmed that splenectomy did not affect the carcinoma cells distribution in liver capillaries via HPV injection (Figure 7d). Importantly, compared to sham‐operated mice inoculated with B16 cells, performed splenectomy significantly reduced liver metastatic tumor burden (Figure 7e,f). The facilitating effect of the spleen in liver metastasis was also observed in another mouse hepatoma cell line, H22‐inoculated VHPV metastasis model (Figure S7c–g, Supporting Information). Considering cGAS inhibitor treatment could block splenocytes infiltration into the liver in VHPV metastasis, cGAS inhibitor treated B16 cells were further inoculated to sham‐operated or splenectomy mice via HPV. Importantly, cGAS inhibitor treatment markedly ameliorated liver metastasis, performed splenectomy did not further alleviate liver metastatic tumor burden in these mice (Figure 7e,f). These results suggest that both cGAS activation and supplementation of splenocytes are essential for liver metastasis. Consistently, cGAS inhibitor treatment showed a less count of CD11b^+^ cells and F4/80^+^ cells infiltration in the metastatic liver compared to mice inoculated with B16 cells. Furthermore, performed splenectomy did not further reduce the count of CD11b^+^ and F4/80^+^ cells in mice inoculated with cGAS inhibitor treatment B16 cells (Figure 7g). Notably, the remaining F4/80^+^ cells in mouse liver were mostly M2‐polarized macrophages (Figure S7h, Supporting Information). To further verify the role of infiltrating macrophages in liver metastasis, macrophages deletion was performed by intravenous inoculation of clodronate liposomal (Figure 7h). A single injection of clodronate liposomal in the tail vein of mice significantly reduced the count of macrophages in the spleen (Figure 7i, Figure S7i, Supporting Information). Compared to directly inoculated B16 cells to mouse liver VHPV, macrophages deletion dramatically ameliorated liver metastatic tumor burden (Figure 7j,k). Taken together, these data suggest that cGAS activation in carcinoma cells and supplementation of splenic monocytes/macrophages to the liver are both essential for exacerbating liver metastasis.

## 3 Discussion

In addition to genetic and external environmental factors, the physical interaction of carcinoma cells with their microenvironment is a key determinant of the metastatic process.^[^ ^53^ ^]^ During their transit pass through the capillaries, carcinoma cells are subjected to hemodynamic forces and mechanical forces.^[^ ^53^ ^]^ However, fully elucidating the influence of physic factors in metastasis remains a big challenge. In this study, we report that in the VHPV liver metastasis, carcinoma cells pass through the narrow capillaries leading to mechanical force‐induced nuclear deformation and cGAS activation. The latter results in robust activation of cGAS‐STING pathway and IL6, TNFα and CCL2 production for recruitment of splenic monocytes/macrophages to liver metastatic niche, which supports metastatic colonialization of carcinoma cells and facilitates liver metastasis. Inhibition of cGAS activation ameliorates VHPV liver metastasis by attenuating pro‐inflammatory cytokine production and monocytes/macrophages infiltration into the liver, however, *Cgas* deletion only results in the decrease of monocytes/macrophages infiltration in the liver, but deletion of adaptor protein *Sting1* results in both the decrease of monocytes/macrophages infiltration and amelioration of VHPV liver metastasis. More importantly, blocking splenocyte migration to the liver by splenectomy has a similar effect to inhibiting cGAS activation, both of which ameliorated VHPV liver metastasis.

The involvement of mechanical force in tumor progression has recently received intensive attention. The cell nucleus has traditionally been considered as a passive storehouse for genetic material, but has now been identified as a mechanosensor of large cell deformation that rapidly converts mechanical inputs into signaling outputs. ^[^ ^54^, ^55^, ^56^ ^]^ In the context of confined migration, mechanical force induces severe nuclear deformation and NE rupture in vitro.^[^ ^57^, ^58^ ^]^ Nuclear deformation has been shown to influence cell differentiation,^[^ ^59^ ^]^ chromatin organization,^[^ ^60^, ^61^, ^62^ ^]^ cell migration,^[^ ^12^, ^13^, ^63^, ^64^ ^]^ and pathfinding in constrained environments.^[^ ^65^ ^]^ Transient NE rupture allows uncontrolled exchange between the nucleoplasm and cytoplasm, which leads to DNA damage ^[^ ^12^, ^13^, ^14^, ^15^, ^20^, ^66^, ^67^ ^]^ and cGAS activation.^[^ ^12^, ^13^, ^15^, ^57^ ^]^ Of note, these results mainly acquire from in vitro study, and what happens during carcinoma cell migration in capillaries is still largely unknown. Here, based on the advancement of intravital microscopy, we directly observed nuclear deformation, NE rupture and cGAS activation of carcinoma cells in liver capillaries. These provided direct evidence for mechanical force‐induced nuclear deformation and cGAS activation in vivo in real time and at single cell resolution.

cGAS activation is poorly tolerated in normal cells, and it often promotes cellular senescence and immune‐mediated clearance.^[^ ^68^, ^69^ ^]^ Paradoxically, cGAS is rarely inactivated in cancer. ^[^ ^70^ ^]^ cGAS activation has been shown to drive cancer progression, metastasis and immunosuppression.^[^ ^14^, ^21^, ^28^, ^30^, ^71^ ^]^ Here, by using a cell compression model in vitro, we observed the production of IL6, TNFα and CCL2 in cGAS‐activated carcinoma cells induced by mechanical force. This supports the hypothesis that mechanical force drives pro‐metastatic niche formation through cGAS‐STING‐CCL2 pathway. Concordantly, a recent study also confirmed that chromosomal instability induced cGAS–STING‐CCL2 pathway promotes downstream signal re‐wiring in cancer cells, which leading to a pro‐metastatic niche formation in lung. ^[^ ^71^ ^]^ Considering that the recruitment and retention of monocytes at metastatic sites is primarily regulated by the CCL2‐CCR2 axis,^[^ ^32^, ^33^, ^34^ ^]^ where they differentiate into macrophages and promote cancer growth.^[^ ^34^ ^]^ The monocytes/macrophages seem to be the principal member of pro‐metastatic niche.^[^ ^71^ ^]^

Furthermore, our results suggest that inhibition cGAS activity and deletion of *Cgas* and *Sting1* block mechanical force‐induced CCL2 production in melanoma VHPV metastasis, thereby attenuating splenic monocytes/macrophages infiltration in the liver. Previously, the spleen has been proven to serve as a monocyte reservoir to accommodate the demands of rapid‐onset inflammation in conditions.^[^ ^40^, ^44^ ^]^ Manipulating cGAS‐STING activation and expression to attenuate splenic monocytes/macrophages infiltration in liver not only occurs at the onset of cancer cell colonization, but also inhibits CD11b^+^ and F4/80^+^ myeloid cell infiltration in metastatic solid tumors. CD11b^+^ and F4/80^+^ myeloid cells are key components of pro‐metastatic niche^[^ ^6^, ^72^ ^]^ and they might be splenic monocytes/macrophages recruited to liver. Their accumulation facilitates carcinoma cell seeding and colonization.^[^ ^49^, ^72^, ^73^ ^]^ Indeed, we observed that inhibition of cGAS in carcinoma cell ameliorates liver metastasis. However, though attenuated inflammatory cell infiltration, *Cgas* knockout does not ameliorate liver metastasis, but *Sting1* knockout ameliorates liver metastasis. This may be due to the nonenzymatic functions of cGAS counteracting the ameliorating effect of cGAS enzyme activity loss in metastasis,^[^ ^35^, ^37^ ^]^ which needs to be further study. Taken together, these findings emphasize the importance of mechanical force and splenocytes, especially splenic monocytes/macrophages, for liver metastasis.

The spleen and liver are tightly connected by HPV. All splenic blood perfuses to the liver via HPV. This is the anatomical infrastructure for the rapid mobilization of splenocyte to the liver. Although the spleen definitely exports splenocyte to liver, and our recent work certified that CD11b^+^CD43^hi^Ly6C^lo^ splenic monocyte migrates into liver to exacerbate fibrosis,^[^ ^44^ ^]^ little knowledge of splenocyte function in liver metastasis is reported. In this study, the migration of splenocyte to liver was blocked by splenectomy and splenic macrophage clearing, and we observed a decrease in CD11b^+^ and F4/80^+^ myeloid cell infiltration and a reduction of metastatic tumor burden in VHPV liver metastasis. This implied a facilitative role of splenic monocytes/macrophages in liver metastasis. Importantly, in our VHPV metastasis model, the carcinoma cell progress to larger solid tumor in 7–10 days, the cytotoxic lymphocyte has not been primed for target carcinoma cell elimination. ^[^ ^74^ ^]^ Thus, the promoting function of splenocyte in liver metastasis mainly performed by myeloid cells, especially splenic monocytes/macrophages. In addition, inhibition of cGAS activity in carcinoma cells attenuates inflammatory cell infiltration and ameliorate VHPV liver metastasis, blocking splenocyte to migrate into liver show a similar effect as the cGAS inhibition. However, combined execution of splenectomy in mice and inhibiting of cGAS activity in carcinoma cells do not further attenuate inflammatory cell infiltration and ameliorate VHPV liver metastasis. Actually, this should represent the underlying mechanism that mechanical force‐induced cGAS activation exacerbates liver metastasis by recruiting splenic monocytes/macrophages to form pro‐metastatic niches in liver.

Nevertheless, it is still unclear how mechanic force induced damaged carcinoma cells interact with these recruited splenic immune cells in vivo and ultimately leads to the effect of facilitating liver metastasis. Our coculture cell experiments have demonstrated that splenic macrophages can promote the survival and proliferation of mechanic force induced damaged carcinoma cells. Generally, immune cells identify and destroy damaged cells to prevent them from causing cancer or other pathologies. However, clinical correlative data and a plethora of studies have shown that TAMs play a cancer promoting role.^[^ ^49^ ^]^ At the metastatic site, metastasis‐associated macrophages promote extravasation, tumor cell survival and persistent growth. TAMs are also suppressive to the activities of cytotoxic T and natural killer cells that have the potential to eradicate tumors.^[^ ^49^ ^]^ A recent work also confirmed that damaged cells generate a p21‐activated chemokines to attract macrophages, these macrophages disengage if cells normalize p21 within 4 days, but if p21 induction persists, they polarize toward an M1 phenotype and lymphocytes mount a cytotoxic T cell response to eliminate target cells.^[^ ^74^ ^]^ This provides a valuable window of time for damaged cells to repair themselves. In damaged carcinoma cell condition, the attracted macrophages appear to eventually polarize toward the M2 phenotype and promote liver metastasis. However, this needs to be further study.

Overall, we provide evidence that mechanical force‐induced cGAS‐STING pathway activation in carcinoma cell facilitates VHPV liver metastasis by recruitment of splenic monocytes/macrophages into liver metastatic niche. Mechanistically, compressed carcinoma cell in capillaries orchestrates this process through activating of cGAS‐STING pathway and the subsequent production of CCL2, which alters the immune microenvironment of the liver by chemoattracting of splenic monocytes/macrophages to establish a pro‐metastatic niche. Critically, our results offer two exciting therapeutic intervention strategies in VHPV liver metastasis. One is intervening of cGAS‐STING‐CCL2 pathway activation by a chemical inhibitor and another is blocking splenic monocytes/macrophages migration into liver metastatic niche, both ameliorated liver metastasis. In conclusion, our work reveals an intriguing link between mechanical challenge, cytosolic DNA sensing pathway, spleen immune cells and liver metastasis. The results emphasize the function of mechanical force in cancer metastasis and provide a practical application for controlling the liver metastasis.

## 4 Conclusion

Although we provide evidence that mechanical force‐induced cGAS activation in carcinoma cell facilitates VHPV liver metastasis by recruitment of splenocyte into liver metastatic niche, and both intervening cGAS‐STING‐CCL2 pathway activation and blocking splenocyte migration into liver metastatic niche ameliorate liver metastasis, further investigation into the cell type of splenocyte and exploration of useful cGAS inhibitor are warranted. Our findings taken together have highlighted the mechanical force roles and splenocytes in liver metastasis, supporting that targeting the mechanic sensor pathway and blocking splenocyte recruitment as candidate therapeutic approaches for liver metastasis.

## 5 Experimental Section

### Cell Lines

The cell lines generated in this study and their parental cell lines (Murine melanoma cells B16, B16^GFP^, B16^GFP/H2B‐RFP^, *Cgas^‐/‐^* B16, B16^cGAS‐GFP/H2B‐RFP^; Murine hepatocellular carcinoma cells H22, H22^GFP^) were grown in RPMI 1640 media with 10% v/v FBS and 100 µg mL^−1^ penicillin/streptomycin. The murine hepatic stellate cells JS1 and cell lines generated in this study (JS1^GFP^, JS1^GFP/H2B‐RFP^, JS1^cGAS‐GFP/H2B‐RFP^) were grown in DMEM media with 15% v/v FBS and 100 µg mL^−1^ penicillin/streptomycin. The murine macrophage cells RAW264.7, normal murine liver cells BNLCL.2 and murine hepatocellular carcinoma cells (Hepa1‐6, Hepa1‐6^cGAS‐GFP^) were grown in DMEM media with 10% v/v FBS and 100 µg mL^−1^ penicillin/streptomycin. The human melanoma cells A375 and 92‐1 were grown in RPMI 1640 media with 10% v/v FBS and 100 µg mL^−1^ penicillin/streptomycin. The human hepatocellular carcinoma cells MHCC‐97H, Hep G2 and the human hepatic stellate cells LX‐2 and LX‐2^GFP/H2B‐RFP^ were grown in DMEM media with 10% v/v FBS and 100 µg mL^−1^ penicillin/streptomycin. Cell cultures at passages 3–8 were used. Cells were passaged or used for in vivo inoculation at 80% confluency.

### Mice

All mice used were on a C57BL/6/N background. Male or female C57BL/6/N mice aged 6–10 weeks were used in animal experiments and purchased from laboratory animal center, Xi'an Jiaotong University. B6. CgGt (ROSA) 26Sor<tm1.1(CAG‐kikGR) Kgwa> (Riken BRC 09256, referred to as KikGR, RRID: IMSR_RBRC09256) mice were purchased from Riken BRC Experimental Animal Division. B6.SJL‐Ptprca Pepcb/BoyJ (no.002014, referred to as CD45.1^+^, RRID: IMSR_JAX:002014) mice were purchased from the Jackson Laboratory. All mice were maintained under specific pathogen‐free controlled conditions and received human care according to the criteria outlined in the NIH Guide for the Care and Use of Laboratory Animals. For tumor studies, male or female animals aged 6–8 weeks were used. Animals were randomly assigned to experimental groups. The investigators were not blinded to allocation during experiments and outcome assessments. In this paper, all mice experiments were completed according to the standard permitted by the Biomedical Ethics Committee of Health Science Center of Xi'an Jiaotong University and used a minimum number of animals (No. XJTUAE2023‐1151).

### Generation of Cgas and Sting1 CRISPR Clones

The guide RNA (gRNA) targeting mouse *Cgas* and *Sting1* were commercially synthesized: g*Cgas* 1, 5′‐GGCGCCGTCGTCCTTCTACG‐(PAM)‐3′; g*Cgas* 2, 5′‐ GCGAGGGTCCAGGAAGGAAC‐(PAM)‐3′; *gSting1* 1, 5′‐ACCTGCATCCAGCCATCCCA‐(PAM)‐3′; *gSting1* 2, 5′‐ GTTGAAAAACCTCTGCTGTC‐(PAM)‐3′. gRNA plasmid was co‐transfected into target cells with an Cas9‐2A‐GFP expression plasmid (Addgene, #172 221) by Lipo3000 (ThermoFisher, Cat# L3000001). 2 × 10^5^ cells were transfected, 1 µg Cas9‐2A‐GFP plasmid, 1 µg g*Cgas* 1 plasmid and 1 µg g*Cgas* 2 plasmid or 1 µg *gSting1* 1 plasmid and 1 µg *gSting1* 2 plasmid were co‐transferred to B16 cells. Cells were then allowed to recover for 24 h before single cell FACs sorting into single wells of a 96 wells plate for single clone amplification and selection. Successful CRISPR/Cas9 editing was confirmed by western blotting of the amplified cell colonies.

### Cell Compression

Cells were compressed using a dynamic confiner similar to previously established planar microconfinement methods.^[^ ^20^ ^]^ To confine cells at different heights, glass beads of either 3, 5, or 10 µm diameter (Invitrogen) were used as spacers to determine the height of compression. Cells were mixed with beads at a density of 1 × 10^6^ µL^−1^ and drop on 24‐mm glass coverslip, then covered another coverslip on the drop and compressed using the device. Coverslips were always cleaned and equilibrated in DMEM before each experiment. Two coverslips separated with microbeads were used (height = 3, 5,10 µm).

### VHPV Metastasis Model

Experimental liver metastasis in mice were performed by implanting 3 × 10^5^ B16 cells or 3 × 10^5^ H22 cells, respectively, unless otherwise stated, in 200 µl PBS into the portal vein of C57BL/6 mice using a 32 G syringe. At the indicated time points, mice were euthanized and metastatic tumor burden was assessed by quantifying liver weight and the frequency and size of metastatic lesions in hematoxylin and eosin‐stained paraffin‐embedded liver sections by microscopy using ZEN imaging software or quantifying GFP labelled cancer cells in liver by FCM analysis. For intravital imaging of carcinoma cells in liver, 1 × 10^6^ B16^GFP^ cells (or B16^GFP/H2B‐RFP^, B16^cGAS‐GFP/H2B‐RFP^, H22^GFP^, JS1^GFP^, JS1^GFP/H2B‐RFP^, JS1^cGAS‐GFP/H2B‐RFP^, LX‐2^GFP^, LX‐2^GFP/H2B‐RFP^, MHCC‐97H, BEL‐7404) were implanting in 200 µl PBS into the portal vein of C57BL/6 mice or KikGR mice using a 32 G syringe. At the indicated time points, mice were anesthetized with ketamine/xylazine and the livers were exposed from the abdominal cavity, and intravital imaging were performed by using the TCS SP8 laser scanning confocal microscope (Leica) or inverted fluorescence microscope (Zeiss). All mice with successful injection were included for further analyses.

### KikGR Photoconversion

KikGR photoconversion was performed as previously described.^[44]^ Briefly, KikGR transgenic mice were anesthetized with ketamine/xylazine, and the spleen was externalized gently. Then sterile aluminum foil was arranged on either side of the spleen to shield the skin and abdominal cavity. The mice were placed within a custom‐designed box, then the spleen was irradiated 5min/field with short wavelength laser light (405 nm) laser under a fluoresce microscope (Zeiss, Germany) equipped with 5 × objective. After photoconversion, the spleen was replaced, and the abdominal cavity and skin were closed.

### Intravital Imaging by Confocal Microscopy

Mice were anesthetized by a mixture of Ketamine/Xylazine and placed within a custom‐designed box. For liver imaging, the surgical preparation of the liver was performed as described previously. ^[^ ^52^, ^75^, ^76^ ^]^ Liver imaging was performed on the inverted TCS SP8 laser scanning confocal microscope (Leica). Alternatively, a compound fluorescence microscope (Zeiss, Germany) was used for some of the liver imaging.

### Propidium Iodide Staining Assay of Carcinoma Cell NE Rupture

For PI labelling of NE ruptured cells in vitro, cells were administered PI (0.5 µg mL^−1^) and hoechst33342 (0.5 µg mL^−1^) in culture medium and incubated at 37 °C in incubator for 20 min. Then, under Zeiss fluoresce microscope, the hoechst33342 stained and PI unstained (nuclear envelop is intact) cells were collected, resuspended with PBS contain 1 µg mL^−1^ PI, and these cells were used to detected ruptured nuclei under cell compression treatment (Figure 2a, Figure S2a–c, Supporting Information). After compression, the cells were imaged directly and immediately on the Zeiss fluoresce microscope.

For PI labelling of NE ruptured cells in liver, the collected cells were resuspended with PBS contain 5 µg ml^−1^ PI, and these cells were inoculated to mouse liver immediately via hepatic portal vein. In 20 min, the mouse liver was performed intravital imaging to detected ruptured nuclei (PI^+^ Hoechst^+^ nuclei) directly on the Zeiss fluoresce microscope (Figure 1j, Figure S1h, Supporting Information).

### Splenectomy

During Ketamine/Xylazine anesthesia, the abdominal cavity of mice was opened and the spleen vessels were cauterized. The spleen was carefully removed and the abdominal cavity was closed. For control experiments, the abdomen was opened, but the spleen was not removed.

### Spleen Transplantation

Spleen transplantation was performed as previously described.^[^ ^44^ ^]^ Briefly, spleen donor mice (CD45.1^+^ or KikGR) were anesthetized and intravenously injected with heparin. Then the donor spleen containing the splenic artery and splenic vein was collected and stored in ice cold heparin‐saline. The spleen of the anesthetized recipient (CD45.2^+^) was removed after ligation of the splenic vein and artery at the splenic hilar. After that, the donor spleen was placed at the right flank of recipient abdomen without twists. End‐to‐side anastomosis was performed on the donor's portal vein to the recipient's portal vein, as well as donor's aortic cuff to the recipient's infrarenal aorta. The abdominal cavity and skin were closed.

### FACS Analysis of Immunocytes in Liver and Metastatic Niches

Mice were perfused with sterile PBS and livers were harvested in PBS with 1% Penicillin/Streptomycin. Briefly, livers were picked, minced, further digested by 0.5 mg mL^−1^ Collagenase IV (Sigma, C4‐BIOC), and 0.001% (w/v) DNase I (D‐4527, Sigma) at 37 °C for 20 min. Collected cells of liver tissues were centrifugated at 50 × g for 5 min to separate of hepatocytes. Then, red blood cells were lysed with RBC lysis reagent (555899, BD Pharmingen) at 4 °C. Then the cells were surface‐labeled with the indicated antibodies for 20 min at 4 °C. Dead cells were excluded by using DAPI staining (Solarbio, 1:1000). The immune cells were incubated with the following antibodies: anti‐mouse CD45 antibody (PerCP/Cy5.5, Biolegend, 103132, 1:200), anti‐mouse CD45.1 FITC (Biolegend, 110705, 1:200), anti‐mouse CD45.2 PE (Biolegend, 109807, 1:500), anti‐mouse CD3 APC (Biolegend, 100236, 1:200), anti‐mouse CD11b BV510 (Biolegend, 101245, 1:200) or BV410 (Biolegend, 101235, 1:200), anti‐mouse F4/80 FITC (Biolegend, 123108, 1:200) or APC (Biolegend, 123116, 1:200) or APC‐Cy7 (Biolegend, 1:200), anti‐mouse Ly6C APC‐Cy7 (Biolegend, 128026, 1:200), anti‐mouse Ly6G PE‐Cy7 (Biolegend, 127618, 1:200) or BV510 (BioLegend, 127618, 1:200). Flow cytometry was performed on a FACSCanto II (BD Biosciences) platform and results were analyzed using FlowJo software or Kaluza Analysis Software. Neutrophils were identified as CD11b^+^Ly6G^+^ cells in the CD45^+^CD3^‐^ gate, inflammatory monocytes were identified as CD11b^+^Ly6C^high^ cells, infiltrating macrophages were identified as CD11b^high^ F4/80^low^ cells in the gate of CD45^+^ live cells, while Kupffer cells were identified as CD11b^low^ F4/80^hi^ cells in the gate of CD45^+^ live cells. Every gate was done on live cells.

### Macrophages Isolation

Primary murine macrophages were isolated from spleen of C57BL/6/N mice or KikGR mice and isolated splenic macrophages were allowed to adhere onto cell culture treated petri dish. After 2 h, cells were washed three times with warm PBS to get rid of non‐macrophage cells. Adherent macrophages were then cultured in complete RPMI for the recruitment assay and adoptive transfusion assays in vivo.

### Clodronate Liposome Treatment

Clodronate liposomal (Clodrosome; 5 mg mL^−1^) were obtained from Encapsula NanoSciences (Brentwood, TN). These liposomal formulations or PBS control were administered to mice as 200 µL injections per mouse (20–40 mg kg^−1^) through tail vein injection. Liposomal formulations injections were administered once 2 days before starting tumor cells inoculation. Depletion was checked by FACS before starting tumor cells inoculation.

### Western Blot

Cultured cells were collected by scraper, then lysed with RIPA buffer on ice for 10 min, followed by centrifugation at 13 000 × *g* for 15 min. The protein concentration was measured with a BCA Protein Assay Kit. Proteins of 10 µg cell lysis were loaded for cGAS, STING, IL6, TNFα, CCL2 and the GAPDH assays, and separated on 10% SDS‐polyacrylamide gel. Thereafter, samples were transferred onto polyvinylidene difluoride (PVDF) membrane (Merck Millipore, IPVH00010). The membrane was blocked for 2 h in 5% nonfat dry milk and incubated with primary antibodies for 2 h. Next, the PVDF membrane were washed three times with PBS containing 0.1% Tween‐20, incubated with HRP‐conjugated secondary antibody for 2 h at room temperature, and then washed with PBS containing 0.1% Tween‐20. Protein bands were visualized using the enhanced chemiluminescence system (Merck Millipore). GAPDH was used as the loading control. Brefeldin A (BD Biosciences, Cat# 347688) was used to block protein transport. The antibodies employed in this study were anti‐cGAS (CST, #31659), anti‐STING (Proteintech, #19851‐1‐AP) and anti‐GAPDH (CST, #2118L), anti‐CCL2 (Proteintech, Cat# 66272‐1‐Ig), anti‐IL6 (CST, Cat# 312912), anti‐TNFα (CST, Cat# 11948), anti‐phospho‐H2A.X(S139) (CST, Cat# 9718).

### qPCR Assay

RNA was isolated, after which cDNA was synthesized using the PrimeScript RT reagent Kit (Takara, RR047A). qPCR was performed on the CFX96 Real Time PCR Detection System (Bio‐Rad) using the TB Green Premix Ex Taq II (Takara, RR820A). Gene expression changes were calculated following normalization to *Actb* using the comparative Ct (cycle threshold) method. The primers used are provided in Table S1 (Supporting Information).

### Apoptosis Analysis

Cells confined at indicated height spaces for indicated time were dissociated by 0.25% trypsin‐EDTA and harvested by centrifugation. Apoptosis was determined using Annexin V Apoptosis Detection Kit (ThermoFisher, #V13245). Briefly, cells were incubated with 100 µL of binding buffer containing 5 µL of FITC‐conjugated Annexin V antibody for 15 min at room temperature. After incubation, cells were washed and resuspended in binding buffer (200 µL) containing 5 µL of PI Staining Solution and analyzed by flow cytometry immediately.

### Immunohistochemistry

Paraffin‐embedded samples were sectioned at 4 µm thickness. For anti‐mouse CD11b, F4/80 and Ki‐67 staining, sections of FFPE liver tissues were dewaxed in xylene and rehydrated in ethanol. Sections were then pretreated in a microwave oven (two cycles for 3 minutes each) in 0.01 M citrate buffer (pH 6.0) to remove aldehyde links formed during initial fixation of tissues. Endogenous peroxidase was blocked for 20 min in H_2_O containing 2% H_2_O_2_. Unspecific sites were blocked in PBS containing 2% bovine serum albumin for 1 h at room temperature and tissues were incubated for two hours with antibody against CD11b (Abcam, ab133357, 1:200), F4/80 (Abcam, Ab300421, 1:200), iNOS (Abcam, Ab115819, 1:100), CD206(Abcam, Ab300621, 1:100) or Ki‐67 (CST, #34330, 1:200) in PBS supplemented with 0.1% BSA. Rat anti‐Mouse HRP IgG (CST, #7074 V) was used as secondary antibody. After washing, slides were developed with DAB and counterstained with Hematoxylin. Tissues were dehydrated with ethanol, mounted and then analyzed with Zeiss macroscopy.

### Two‐chamber Cell Migration Assays

The procedure of two‐chamber migration assays was performed. Briefly, 5  × 10^5^ freshly isolated splenic macrophages or RAW264.7 cells in RPMI 1640 were added to the upper chamber (363096, BD), and a 2 × 10^5^ B16 cells or CM of B16 cells was added to the lower chamber as the chemoattractant. The migrated cells in the lower chamber were counted at indicated time.

### Splenocytes Adoptive Transfer

Splenocytes were isolated from the spleen of CD45.1^+^ C57BL/6 mice or KikGR mice. Isolated splenocytes purity was confirmed by flow cytometry. Splenocytes (2 × 10^6^ cells in 100 µL of PBS) or PBS (100 µL) were injected intravenously into CD45.2^+^ recipient mice.

### Transwell migration

Migration assays used 24‐well inserts with 5‐µm‐pore filters. Cells were first detached and then seeded on top of a Transwell membrane (Corning) at a density of 3 × 10^5^ cells cm^−2^. Medium supplemented with 10% FBS and 1% penicillin/streptomycin was added to the bottom of the membrane. Transwell membranes were imaged by fluorescence microscope to observe nuclear deformation and NE rupture.

### Cell Size, Nuclear Size, and Nuclear Roundness Assays

Cells and nuclear diameter measurements were calculated with Fiji (Measure tool, Fiji) from GFP expression, hochest33342 stained fluorescence microscope images of cell nuclei and Lamin A/C‐stained immunofluorescence microscope images of cultured cells and cells in liver frozen sections. The nuclear roundness index is simply defined as the length of the cell nucleus on minor axis / the length of the cell nucleus on major axis.

### cGAMP Quantification

For intracellular cGAMP quantification, cancer cells were seeded in 10 cm culture dishes. The culture plates were 80% confluent, media were changed to serum‐free and phenol red–free RPMI (Corning). Sixteen hours later, cells were washed with PBS twice and then trypsinized for 1 min at 37 °C, and cell counts were measured. Cells were then centrifuged at ≥600 × *g* at 4 °C for 15 min. Whole cell lysates were generated by lysing the cell pellet in RIPA lysis buffer. The homogenate was then subjected to centrifugation at 10000 *g* for 15 minutes. All the steps were performed on ice. cGAMP ELISA was performed according to the manufacturer's protocol using 2′3'‐Cyclic GAMP ELISA Kit (ThermoFisher/EIAGAMP).

### Statistical Analyses

All data are shown as mean ±s.d.; Statistical significance among groups were evaluated with two‐sided unpaired student's t‐test using GraphPad Prism software. For multiple group comparison, Dunnett's multiple comparison test (one‐way ANOVA) was performed. A probability value of *p* < 0.05 was considered significant. The in vitro experiments were repeated independently at least three times with similar results, as indicated in the Figure legends. For animal experiments, *n* = 4–8 mice per group. Significance was set at *p* < 0.05 (*p < 0.05, **p < 0.01, ***p < 0.001).

### Data and Code Availability

Oligonucleotide sequences used in this study are provided in Supporting Information Table S1. All data are available upon request. Further information and requests for resources and reagents are available from the corresponding author on reasonable request.

**Key Resources Table**

**Table:** 

reagent or resource	source	identifier
**Antibodies**		
GAPDH (for WB 1:10 000)	CST	Cat# 2118L, RRID: AB_561053
cGAS (for WB 1:1000)	CST	Cat# 31659, RRID: AB_2799008
STING (for WB 1:2000)	Proteintech	Cat# 19851‐1‐AP, RRID: AB_10665370
CCL2 (for WB 1:2000)	Proteintech	Cat# 66272‐1‐Ig, RRID: AB_2861337
IL6 (for WB 1:1000)	CST	Cat# 12912, RRID: AB_279805
TNFα (for WB 1:1000)	CST	Cat# 11948, RRID: AB_2687962
phospho‐H2A.X (for WB 1:1000)	CST	Cat# 9718, RRID: AB_2118009
iNOS (for IHC/IF 1:100)	Abcam	Cat# ab115819, RRID: AB_10898933
CD206 (for IHC/IF 1:100)	Abcam	Cat# ab300621, RRID: AB_2935881
F4/80 (for IHC 1:200)	Abcam	Cat # ab300421, RRID: AB_2936298
CD11b (for IHC 1:200)	Abcam	Cat # ab133357, RRID: AB_2650514
Lamin A/C (for IF 1:500)	Proteintech	Cat # 10298‐1‐AP, RRID:AB_2296961
Ki‐67(for IHC 1:200)	CST	Cat# 34330, RRID: AB_2942026
HRP‐conjugated Rat Anti‐mouse IgG	CST	Cat# 7074 V, RRID: AB_2099233
HRP‐conjugated Goat Anti‐rabbit IgG	Millipore	Cat# 401315, RRID: AB_2617117
CD11b (FACS analyses)	Biolegend	Cat#101245, RRID: AB_2561390; Cat#101235, RRID: AB_11203704
CD45(FACS analysess)	Biolegend	Cat#103132, RRID: AB_893344
CD45.1(FACS analysess)	Biolegend	Cat#110705, RRID: AB_313495
CD45.2(FACS analysess)	Biolegend	Cat#109807, RRID: AB_313445
Ly6C (FACS analysess)	Biolegend	Cat#128026, RRID: AB_10640120
Ly6G (FACS analyses)	Biolegend	Cat#127618, RRID: AB_1877262; Cat#127633, RRID: AB_2562937
F4/80(FACS analyses)	Biolegend	Cat#123108, RRID: AB_893502; Cat#123116, RRID: AB_893481; Cat#123117, RRID: AB_893489
CD3ε	Biolegend	Cat#100236, RRID: AB_2561456
**Chemicals, peptides, and recombinant proteins**		
Collagenase IV	Sigma	Cat# C4‐BIOC
DNase I	Sigma	Cat# D‐4527
RU.521	MCE	Cat# HY‐114180
Brefeldin A	BD Biosciences	Cat# 347688
DAPI	Solarbio	Cat# 28718‐90‐3
Hochest33342	Solarbio	Cat# 23491‐52‐3
Propidium Iodide	ThermoFisher	Cat# V13245
Alexa Fluor™ 488‐ conjugated Annexin V	ThermoFisher	Cat# V13245
Lipo3000	ThermoFisher	Cat# L3000001
Clodronate liposomal	Clodrosome	Cat# CLD‐8901
RIPA	Beyotime	Cat# P0013C
RBC lysis reagent	Beyotime	Cat# C3702
Protease inhibitor cocktail	Merck	Cat# 539134
BBS1	NEB	Cat# R0539V
T7 DNA Ligase	NEB	Cat# M0318S
T4 PNK	Sangon Biotech	Cat# B300053‐0500
**Critical commercial assays**		
2′3'‐ Cyclic GAMP ELISA Kit	ThermoFisher	Cat#EIAGAMP
PrimeScript RT reagent Kit	Takara	Cat#RR047A
TB Green Premix Ex Taq II	Takara	Cat#RR820A
**Oligonucleotides**		
Mouse cGAS knockout, qPCR primers, reverse‐transcription primers	This paper	Table S 1
**Experimental models: cell lines**		
B16 and its sublines	ATCC	N/A
H22 series	ATCC	N/A
JS1 series	ATCC	N/A
Hepa1‐6	ATCC	N/A
BNL‐CL2	ATCC	N/A
LX‐2	ATCC	N/A
A375	ATCC	N/A
92‐1	ATCC	N/A
MHCC‐97H	ATCC	N/A
HepG2	ATCC	N/A
HT1080	ATCC	N/A
BEL‐7404	ATCC	N/A
**Software and algorithms**		
FiJi software	https://imagej.net/Fiji/Downloads	RRID: SCR_002285
FlowJo	FlowJo LLC	https://www.flowjo.com/solutions/flowjo; RRID: SCR_008520
Kaluza Analysis Software	Beckman Coulter	https://www.mybeckman.cn/flow‐cytometry/software/kaluza/b16407
Origin 8.5	OringinLab	https://www.originlab.com

## Conflict of Interest

The authors declare no conflict of interest.

## Author Contributions

X.Z. and N.H. contributed equally to this work. X.Z., S.L., Z.L. conceptualized this study. X.Z. performed most of the experiments. N.H. performed the immune‐histochemical experiments. H.C. performed the confocal intravital imaging of mouse livers. Y.M., P.L., J.S. helped to generate gene knockout cell lines and engineering fluorescent protein labeled cells. M.Z., S.Z., H.Z. performed the animal surgery like splenectomy, splenocytes adoptive transfusion and spleen transplantation. H.D., K.F., L.Y., helped with qPCR and western blotting analyses. Q.S., X.L., C.Z., M.S. helped with cell sorting and flow cytometry. G.K., J.G., helped interpret the data. X.Z., S.L., generated the Figures and wrote the manuscript. Z.L. designed and supervised the study. All authors discussed the results and approved the manuscript.

## Supporting information



Supporting Information

## Data Availability

The data that support the findings of this study are available from the corresponding author upon reasonable request.
