# Supplementary material for: Mechanical Force‐Induced cGAS Activation in Carcinoma Cells Facilitates Splenocytes into Liver to Drive Metastasis
Source: Adv Sci (Weinh). 2024 Dec 31;12(8):2401127. doi: 10.1002/advs.202401127 (PMC11848607; doi:10.1002/advs.202401127)
Supplement: Supplementary file 1 — Supporting Information [file ADVS-12-2401127-s001.docx]

Supporting Information

**Title: Mechanical Force-induced cGAS Activation in Carcinoma Cells Facilitates Splenocytes into Liver to Drive Metastasis**

**Authors:** *Xurui Zhang, Na Huang, Yanhua Mu, Haiyan Chen, Mengchen Zhu, Shaoying Zhang, Pengfei Liu, Hailong Zhang, Huan Deng, Keping Feng, Qi Shang, Xi Liu, Chen Zhang, Mengjiao Shi, Lan Yang, Jin Sun, Guangyao Kong, Jing Geng, Shemin Lu*, Zongfang Li**


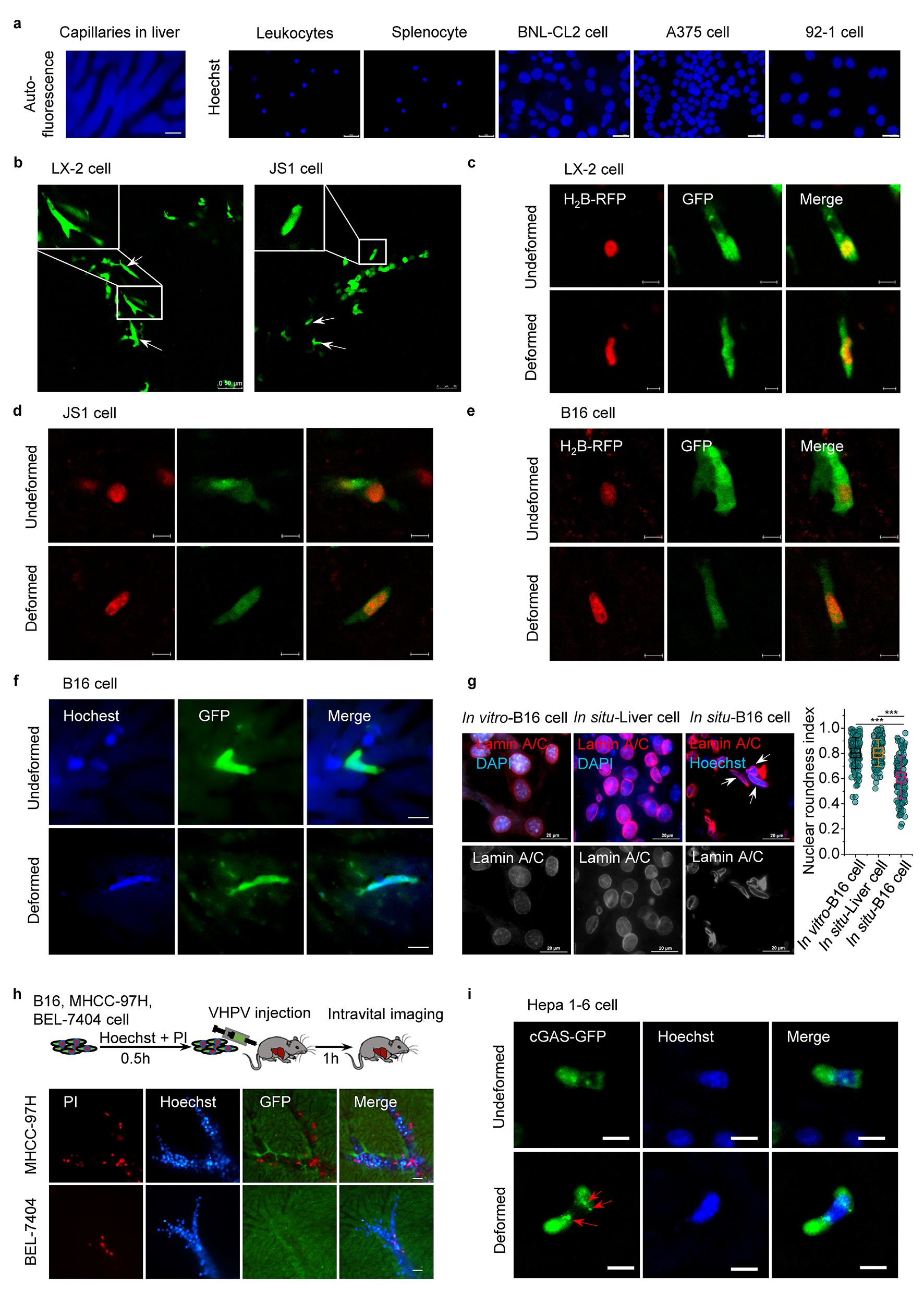


**Figure S1. Nuclear deformation and cGAS activation occur in different cell lines in VHPV inoculation by intravital imaging.** **Related to Figure 1**

(**a**) Representative fluorescence images of the nuclei of mouse leukocyte, splenocyte, hepatocyte (BNL-CL2 cell) and human A375 and 92-1 melanoma cells and intravital images of liver capillaries in GFP mice. Nuclei were stained with DAPI. Scale bars, 20 μm. (**b**) Representative confocal intravital images of LX-2^GFP^ cell (LX-2 cells stably expressing GFP, LX-2^GFP^) and JS1^GFP^ cell (JS1 cells stably expressing GFP, JS1^GFP^) deformation in liver capillaries of C57BL/6 mice. Scale bars, 20 μm. (**c-e**) Representative confocal intravital images of (**c**) LX-2^GFP/H2B-RFP^ cell (LX-2 cell stably expressing GFP and H_2_B-RFP), (**d**) JS1^GFP/H2B-RFP^ cell (JS1 cell stably expressing GFP and H_2_B-RFP) and (**e**) B16^GFP/H2B-RFP^ cell (B16 cell stably expressing GFP and H_2_B-RFP) exhibited nuclear deformation in liver capillaries of C57BL/6 mice VHPV inoculation. Scale bars, 20 μm. (**f**) Representative intravital images of deformed nuclei of B16 cells in the liver capillaries of C57BL/6 mice. B16^GFP^ cell stained with hochest33342 were inoculated VHPV of mice and the liver were imaged at 6 h. Scale bars, 20 μm. (**g**) Immunofluorescence analysis of lamin A/C (red in merged channels and gray in single channels), DAPI (blue), and Hoechst 33342 (blue). Scale bars, 20 μm. (**h**) Representative fluorescence intravital images of MHCC-97H and BEL-7404 human hepatic cancer cell nuclei in liver capillaries of C57BL/6 mice. C57BL/6 mice were injected with cell lines stained with Hoechst (DNA, blue) and co-incubation with PI via HPV and intravital imaged the liver at 30 min. Data shown ruptured nuclei (Red, PI^+^) in liver capillaries. scale bars, 50 μm. (**i**) Representative confocal intravital images of Hepa1-6 cells expressing cGAS-GFP in liver capillaries of C57BL/6 mice. Data shown cGAS-GFP foci (red arrows point to) formation in liver. Scale bars, 10 μm.


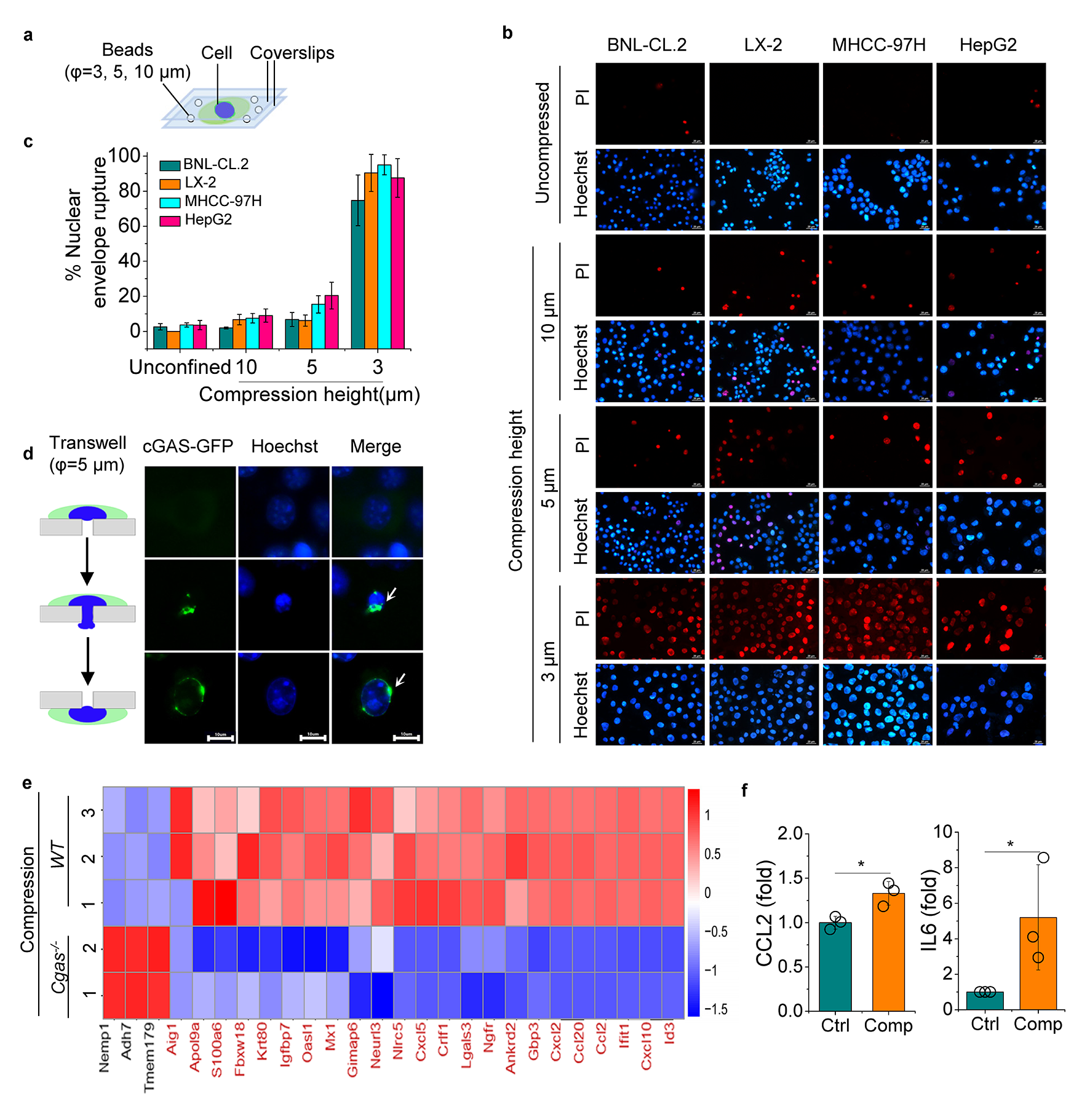


**Figure S2. Nuclear deformation, NE rupture and cGAS activation occurred in either cell compression and confined migration.** **Related to Figure 2**

(**a**)Schematic of the custom-built nuclear compression device. Polystyrene beads serve as spacers to ensure a uniform height between the two glass coverslips. Inset shows cells compressed between the two coverslips. (**b**) Representative fluorescence images of the nucleus of multiple cell lines (mouse hepatocytes BNL-CL.2, human hepatic stellate cell LX-2 and human hepatoma MHCC-97H and HepG2 cells) under compression. Cells were stained with Hoechst (DNA, blue) and PI(Red) and compressed under different height (3,5,10 μm). PI-stained nuclei (Red) indicated NE rupture in nuclear compression. Scale bars, 20 μm. (**c**) Quantification of NE rupture cells during nuclear compression are shown. Mean ± s.d., Data are representative of three independent experiments. (**d**) Representative fluorescence images of the nuclear and cGAS-GFP foci formation in B16^cGAS-GFP^ cell stained with Hoechst (DNA, blue) migrated through the hole (dimeter 5 μm) on the transwell insert. Scale bars, 10μm. (e) Heatmaps of RNA-seq data depicting cGAS-dependent up-regulated genes induced by cell compression treatment of B16 cells. (f) ELISA analysis of inflammatory and cytokines in supernatant of carcinoma cells at 24 h under control or cell compression.


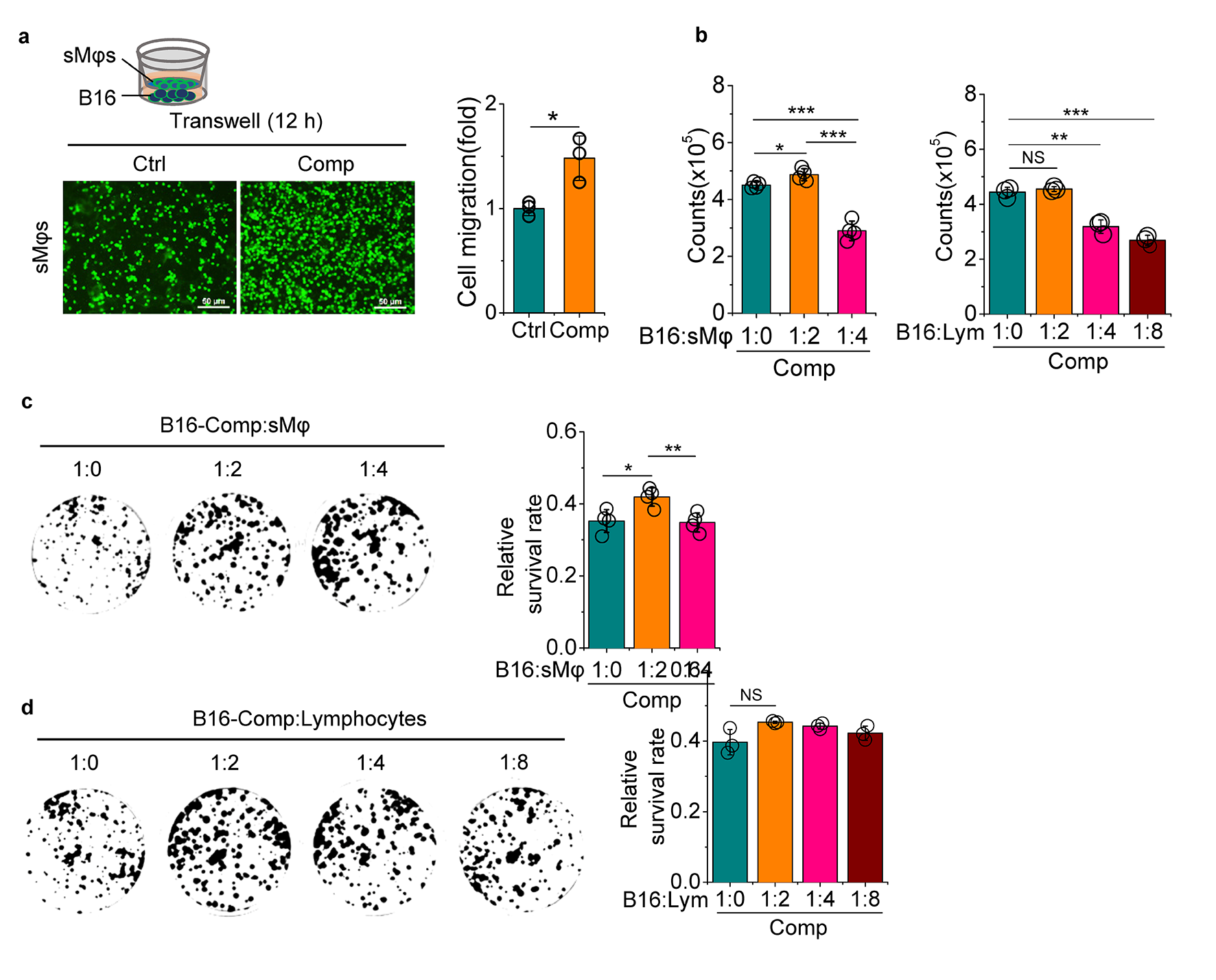


**Figure S3. Splenic macrophage improves the proliferation and survival of compressed carcinoma cell. Related to Figure 3**

(**a**) Migration of splenic macrophages recruited by B16 cells under control and compressed. Scale bar, 50 μm. (**b**) Proliferation rates of compressed B16 cells were determined by coculture with splenic macrophage or splenic lymphocyte at indicated ratio. Scale bar, 20 μm. (**c)** Clonogenic assay to detect colony formation of compressed B16 cells co-culture with splenic macrophage at indicated ratio. (**d**) Clonogenic assay to detect colony formation of compressed B16 cells co-culture with splenic lymphocyte at indicated ratio.


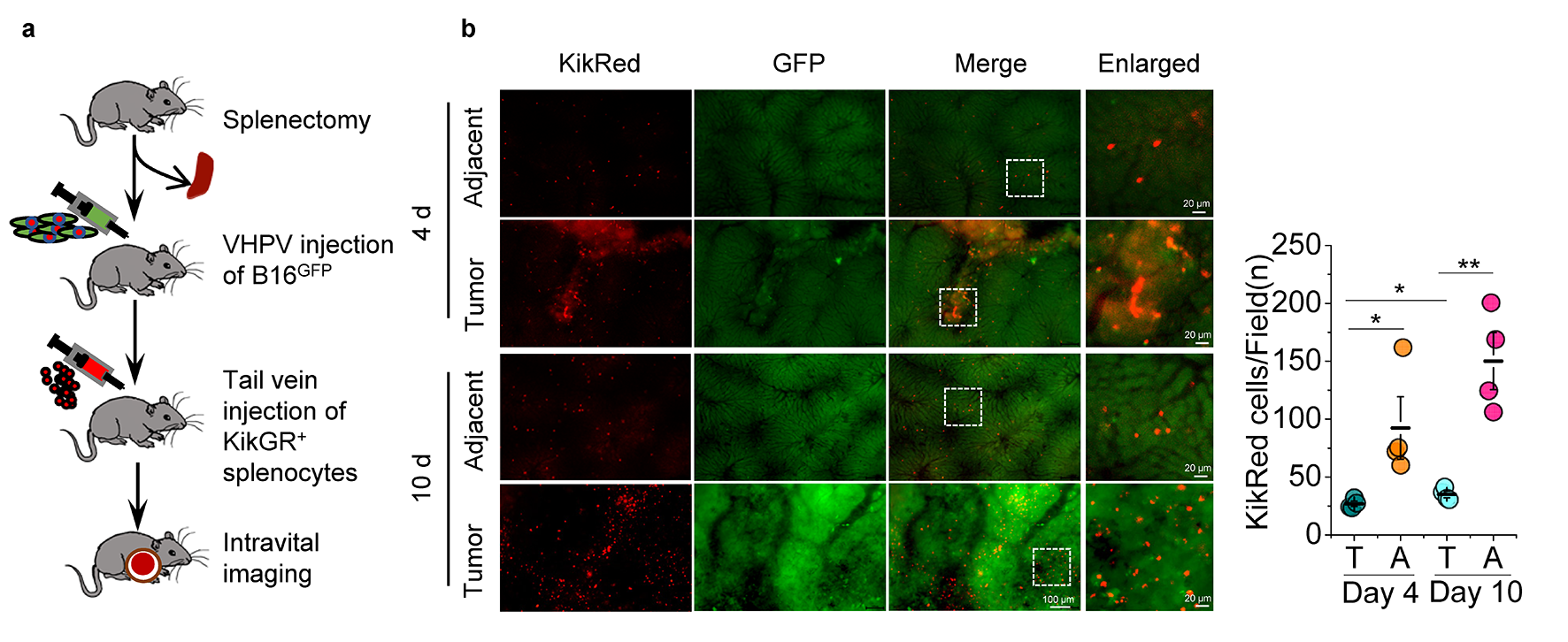


**Figure S4. Splenic monocytes/macrophages infiltrate to metastatic niche in VHPV metastasis. Related to Figure 4**

(**a**) Scheme of splenic cell adoptive transfusion assays. Splenectomy was performed in C57BL/6 mice, and then 3×10^5^ B16^GFP^ cells were injected VHPV of these mice.12 hours later, a total of 5×10^6^ splenic CD45^+^CD11b^+^ cells sorted from KikGR mice were adoptively transferred to these mice by tail vein injection, (**b**) Representative fluorescence intravital images of splenocytes infiltration (KikRed^+^) and metastatic melanoma (GFP^+^) in mouse liver at indicated time point after tail vein injection of splenocytes. Graphical quantification showing the count of KikRed^+^ cells in adjacent and tumor area in liver. (n=4 mice, mean ± s.d.), two-tailed unpaired Student’s t-test were performed for the statistical significance. * and ** stand for p<0.05, <0.01, respectively.


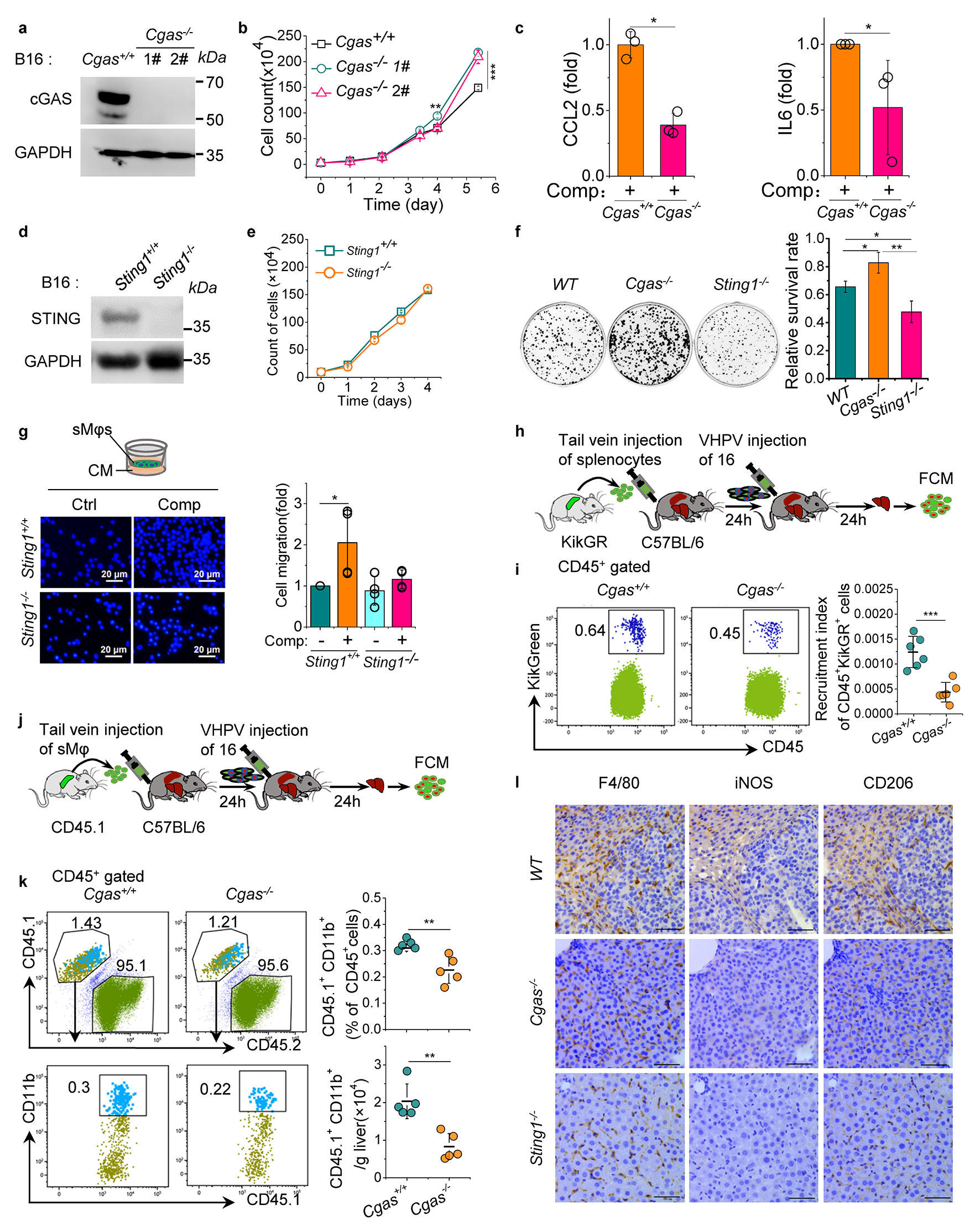


**Figure S5. cGAS knockout enhances carcinoma cell proliferation and attenuates inflammatory cell infiltration.** **Related to Figure 5**

(**a**) Immunoblots for cGAS of *Cgas^+/+^* and *Cgas^-/-^* B16 cells with Gapdh as a loading control. (**b**) Thirty-thousand cells were seeded into each well of a six-well plate. The number of *Cgas^+/+^* B16 or *Cgas^-/-^* B16 cells was counted at indicated timepoints until reaching high density. (**c**)ELISA analysis of inflammatory and cytokines in supernatant of *Cgas^+/+^* and *Cgas^-/-^* B16 cells at 24 h under cell compression. (**d**) Immunoblots for STING of *Sting^+/+^* and *Sting^-/-^* B16 cells with Gapdh as a loading control. (**e**) Thirty-thousand cells were seeded into each well of a six-well plate. The number of *Sting^+/+^* and *Sting^-/-^* B16 cells cells was counted at indicated timepoints until reaching high density. (**f**) Clonogenic assay to detect colony formation of wild type, *Cgas^-/-^* or *Sting^-/-^* B16 cells. (**g**) Migration of splenic macrophages (sMφs) recruited by *Sting^+/+^* and *Sting^-/-^* B16 cells under control or compressed. Scale bars, 20 μm. (**h**) Scheme of splenocytes adoptive transfusion assays. (**i**) The KikGR^+^CD45^+^ splenocytes was detected by FCM in *Cgas^+/+^* and *Cgas^-/-^* B16 cells inoculated C57BL/6 mouse liver. Graphical quantification showing the recruitment index of KikGreen^+^ CD45^+^ splenocytes infiltration in *Cgas^+/+^* and *Cgas^-/-^* B16 cells inoculated mice. Recruitment index: frequency of target cells in the recipient liver/frequency of target cells in transfused splenocytes. (n=6 mice, mean ± s.d.). (**j**) Scheme of splenocytes adoptive transfusion assays. (**k**) The CD45.1^+^ CD11b^+^ splenocyte was detected by FCM in *Cgas^+/+^* and *Cgas^-/-^* B16 cells inoculated CD45.2^+^ mouse liver. Graphical quantification showing the difference of CD45.1^+^ CD11b^+^ splenocyte infiltration in *Cgas^+/+^* and *Cgas^-/-^* B16 cells inoculated mice. (n=5 mice, mean ± s.d.). (**l**) Representative histological images of iNOS^+^ and CD206^+^ cell staining for M1(iNOS^+^) and M2 (CD206^+^) type macrophages determination in serial sections of paraffin-embedded liver tissue from wild type B16 cells, *Cgas^-/-^* and *Sting^-/-^* B16 cells inoculated mice. Scale bars, 20μm. Data shown in (b) and (d-f) are representative of three independent experiments. Mean ± s.d., two-tailed unpaired Student’s t-test were performed for the statistical significance. *, **, and *** stand for p<0.05, <0.01, and <0.001, respectively.


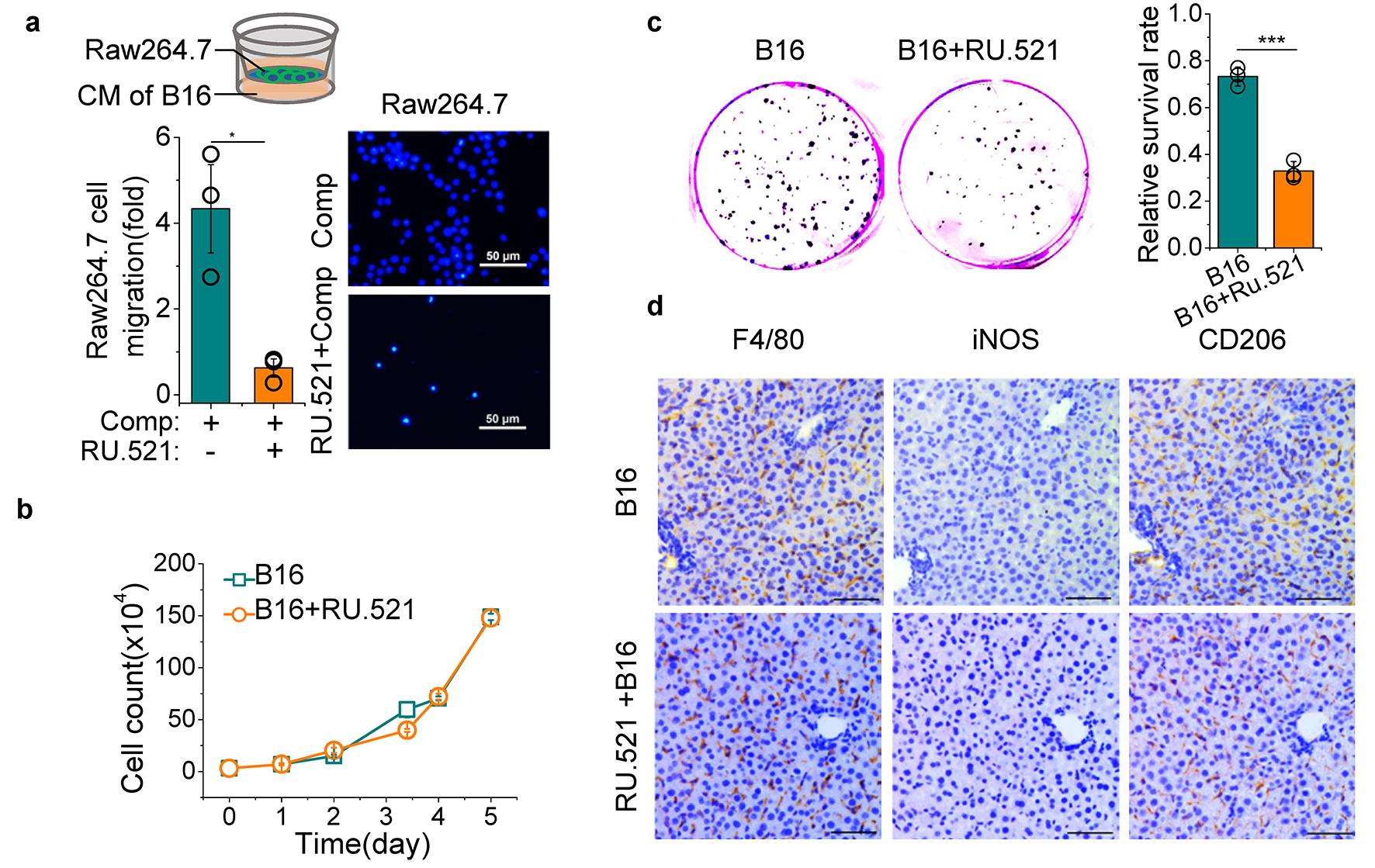


**Figure S6. Inhibition of cGAS activity suppresses cell survival. Related to Figure 6**

(**a**) Migration of Raw264.7 cells recruited by CM of B16 cells under control and compressed in the presence or absence of RU.521. Scale bars, 50 μm. CM: culture medium. (**b**) Thirty-thousand cells were seeded into each well of a six-well plate. The number of B16 cells treated with or without of RU.521 was counted at indicated timepoints until reaching high density. (**c**) Clonogenic assay to detect colony formation of B16 cells treated with or without RU.521. (**d**) Representative histological images of iNOS^+^ and CD206^+^ cell staining in serial sections of paraffin-embedded liver tissue from B16 cells and RU.521 treatment B16 cells inoculated mice. Scale bars, 20μm.


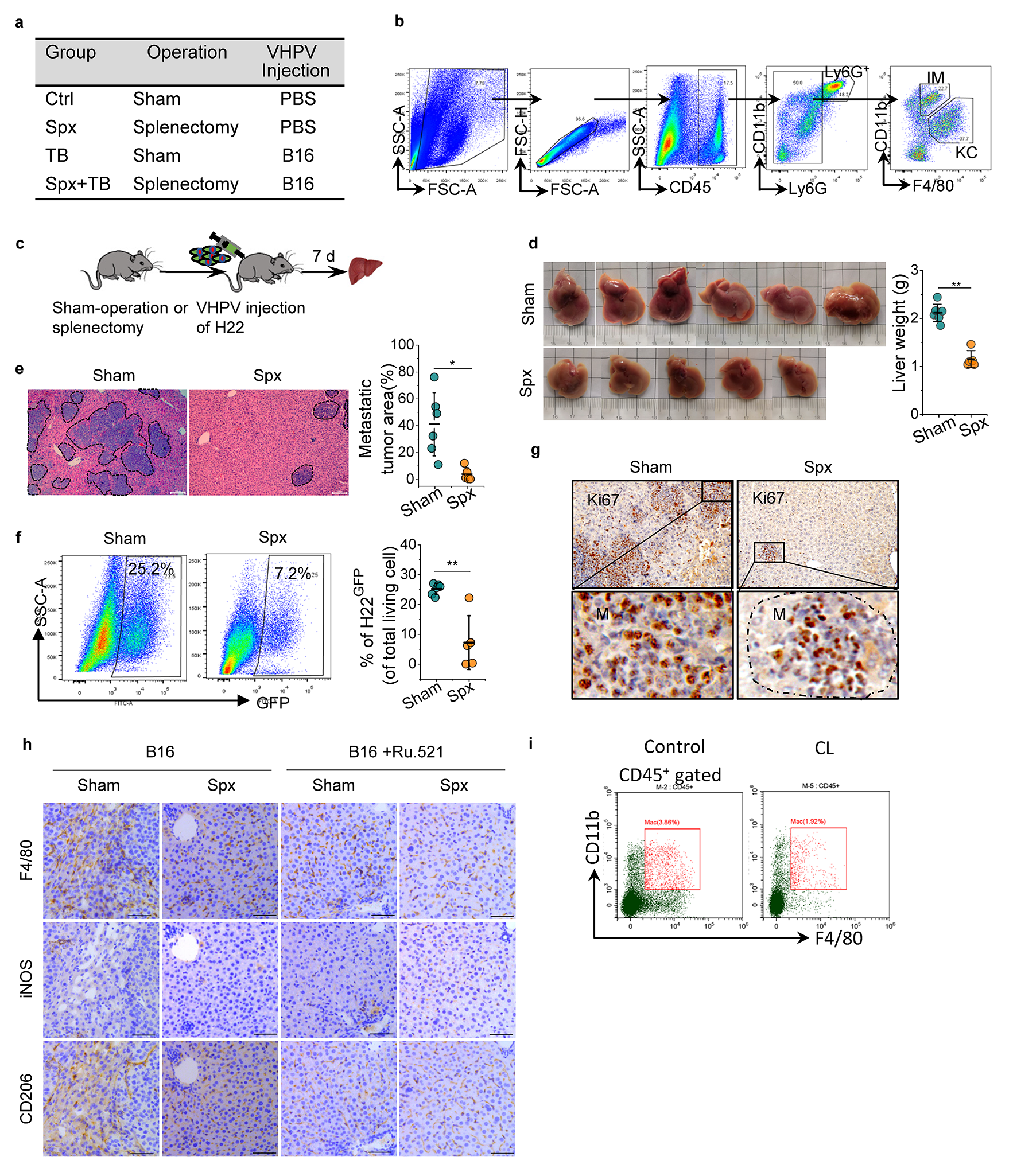


**Figure S7. Spleen plays an essential role in VHPV metastasis. Related to Figure 7**

(**a**) The mice experiment grouping for investigating the function of spleen in myeloid cells accumulation in liver metastatic niche. (**b**) Representative cytometry for gating neutrophils (CD45^+^CD11b^hi^Ly6G^+^), Kupffer cells(CD45^+^Ly6G^-^CD11b^low^ F4/80^hi^) and infiltrating macrophages （CD45^+^Ly6G^-^CD11b^hi^ F4/80^low^）from mouse livers inoculated with B16 cells. (**c**) The experimental scheme for investigating the function of spleen in liver metastasis by using mouse hepatoma cell line H22. (**d**) Representative images showing metastatic tumor burden on the liver surface of mice performed sham-operation or splenectomy and inoculated with H22 cells stably expressing GFP VHPV injection. Graphical quantification showing the weight of livers. (n=5-6 mice, mean ± s.d.). (**e**) Representative images showing tumor area in HE-stained liver sections of (**d**), Graphical quantification showing the percentage of metastatic tumor area in HE-stained liver sections. Scale bars, 100 μm. (**f**) Representative images showing the percentage of H22^GFP^ cells in livers by FCM detecting. Graphical quantification showing the difference of the percentage of H22^GFP^ cells in liver of mice performed sham-operation or splenectomy and inoculated with H22^GFP^ cells VHPV injection. (**g**) Representative images showing the cell proliferation markers (Ki67) staining using paraffin liver sections 7 days after H22^GFP^ inoculation. Scale bars, 100 μm. (**h**) Representative histological images of iNOS^+^ and CD206^+^ cell staining in serial sections of paraffin-embedded liver tissue from indicated mice groups. Scale bars, 20μm. (**i**) Representative flow cytometry dot plots showing a marked reduction of macrophages (CD11b^+^F4/80^+^) in spleen of mice treated with Clodronate Liposomes (CL) compared to control mice treated with PBS Liposomes (Control) (n= 6 mice per condition; one experiment). Spx, splenectomy; Sham, Sham-operation. Data are shown mean ± s.d., two-tailed unpaired Student’s t-test were performed for the statistical significance. *, **, and *** stand for p<0.05, <0.01, and <0.001, respectively.

| **Table S1. Oligonucleotides used in the study, Related to Methods.** | | |
| --- | --- | --- |
| For knockout | Cgas-sg-1F | CACCGGGCGCCGTCGTCCTTCTACG |
|  | Cgas-sg-1R | AAACCGTAGAAGGACGACGGCGCCC |
|  | Cgas-sg-2F | CACCGGCGAGGGTCCAGGAAGGAAC |
|  | Cgas-sg-2R | AAACGTTCCTTCCTGGACCCTCGCC |
| qPCR primers | Mouse-Irf3-F | GAGAGCCGAACGAGGTTCAG |
|  | Mouse-Irf3-R | CTTCCAGGTTGACACGTCCG |
|  | Mouse-Ifit2-F | AGTACAACGAGTAAGGAGTCACT |
|  | Mouse-Ifit2-R | AGGCCAGTATGTTGCACATGG |
|  | Mouse-Cxcl10-F | CCAAGTGCTGCCGTCATTTTC |
|  | Mouse-Cxcl10-R | GGCTCGCAGGGATGATTTCAA |
|  | Mouse-Ccl2-F | TAAAAACCTGGATCGGAACCAAA |
|  | Mouse-Ccl2-R | GCATTAGCTTCAGATTTACGGGT |
|  | Mouse-Ccl5-F | GCTGCTTTGCCTACCTCTCC |
|  | Mouse-Ccl5-R | TCGAGTGACAAACACGACTGC |
|  | Mouse-Ccl8-F | TCTACGCAGTGCTTCTTTGCC |
|  | Mouse-Ccl8-R | AAGGGGGATCTTCAGCTTTAGTA |
|  | Mouse-Cxcl1-F | CTGGGATTCACCTCAAGAACATC |
|  | Mouse-Cxcl1-R | CAGGGTCAAGGCAAGCCTC |
|  | Mouse-Cxcl2-F | CCAACCACCAGGCTACAGG |
|  | Mouse-Cxcl2-R | GCGTCACACTCAAGCTCTG |
|  | Mouse-Cxcl5-F | GTTCCATCTCGCCATTCATGC |
|  | Mouse-Cxcl5-R | GCGGCTATGACTGAGGAAGG |
|  | Mouse-Il6-F | TAGTCCTTCCTACCCCAATTTCC |
|  | Mouse-Il6-R | TTGGTCCTTAGCCACTCCTTC |
|  | Mouse-Tnf-F | CCCTCACACTCAGATCATCTTCT |
|  | Mouse-Tnf-R | GCTACGACGTGGGCTACAG |
|  | Mouse-Cgas-F | GAGGCGCGGAAAGTCGTAA |
|  | Mouse-Cgas-R | TTGTCCGGTTCCTTCCTGGA |
|  | Mouse-STING-F | GGTCACCGCTCCAAATATGTAG |
|  | Mouse-STING-R | CAGTAGTCCAAGTTCGTGCGA |
|  | Mouse-Actb-F | GGCTGTATTCCCCTCCATCG |
|  | Mouse-Actb-R | CCAGTTGGTAACAATGCCATGT |
| For reverse transcription of miRNA | RT-primer | CAGGTCCAGTTTTTTTTTTTTTTTTTVN; V, A/C/G; N, A/G/C/T. |
